# m6A-related lncRNAs predict prognosis and indicate immune microenvironment in acute myeloid leukemia

**DOI:** 10.1038/s41598-022-05797-5

**Published:** 2022-02-02

**Authors:** Fangmin Zhong, Fangyi Yao, Ying Cheng, Jing Liu, Nan Zhang, Shuqi Li, Meiyong Li, Bo Huang, Xiaozhong Wang

**Affiliations:** 1 Jiangxi Province Key Laboratory of Laboratory Medicine, Department of Clinical Laboratory, The Second Affiliated Hospital of Nanchang University, No. 1 Minde Road, Nanchang, 330006 Jiangxi China; 2 School of Public Health, Nanchang University, No. 461 BaYi Boulevard, Nanchang, 330006 Jiangxi China

**Keywords:** Cancer, Computational biology and bioinformatics, Immunology, Molecular biology

## Abstract

Acute myeloid leukemia (AML) is a complex hematologic malignancy. Survival rate of AML patients is low. *N*6-methyladenosine (m^6^A) and long non-coding RNAs (lncRNAs) play important roles in AML tumorigenesis and progression. However, the relationship between lncRNAs and biological characteristics of AML, as well as how lncRNAs influence the prognosis of AML patients, remain unclear. In this study. In this study, Pearson correlation analysis was used to identify lncRNAs related to m^6^A regulatory genes, namely m^6^A-related lncRNAs. And we analyzed their roles and prognostic values in AML. m^6^A-related lncRNAs associated with patient prognosis were screened using univariate Cox regression analysis, followed by systematic analysis of the relationship between these genes and AML clinicopathologic and biologic characteristics. Furthermore, we examined the characteristics of tumor immune microenvironment (TIME) using different IncRNA clustering models. Using LASSO regression, we identified the risk signals related to prognosis of AML patients. We then constructed and verified a risk model based on m^6^A-related lncRNAs for independent prediction of overall survival in AML patients. Our results indicate that risk scores, calculated based on risk-related signaling, were related to the clinicopathologic characteristics of AML and level of immune infiltration. Finally, we examined the expression level of TRAF3IP2-AS1 in patient samples through real-time polymerase chain reaction analysis and in GEO datasets, and we identified a interaction relationship between SRSF10 and TRAF3IP2-AS1 through in vitro assays. Our study shows that m^6^A-related lncRNAs, evaluated using the risk prediction model, can potentially be used to predict prognosis and design immunotherapy in AML patients.

## Introduction

Acute myeloid leukemia (AML) is a type of hematologic malignancy characterized by malignant proliferation of immature bone marrow stem cells in the bone marrow and peripheral blood. AML is highly heterogenous and extremely invasive. The French–American–British (FAB) classification divides AML into eight types: M0–M7^1^. The pathogenesis of AML is still unclear. Numerous studies have examined factors affecting the tumorigenesis and progression of AML from the perspectives of molecular genetics, immunophenotyping, and profiling of gene-expression^2^, and genomic^3,4^ and epigenetic mutations^5,6^. These mutations prevent the differentiation of bone marrow hematopoietic stem cells (HSCs)/progenitor cells and produce self-renewing leukemia cells, leading to generation of malignant clones of myeloid cells. Delineating the pathogenesis of AML has led to new therapies, such as FLT3 mutation inhibitors^7^ and epigenetic therapy of TP53 mutations^8^, that have improved the quality of life for AML patients^9^. AML can occur at any age. The incidence of AML increases with age, and it is most commonly observed in the elderly^10^. Treating AML is challenging because it is a highly heterogenous malignancy. Standard treatments, such as chemotherapy or HSC transplantation (HSCT), are usually used after induction therapy according to the individual characteristics of the patient^11^. However, the survival rate of AML patients treated using standard regimens remains low. Recurrence and related complications are the most common causes of death in AML patients^12^. AML recurrence is often characterized by new mutations that are resistant to chemotherapy^13^. The molecular complexity of AML warrants further studies aimed at investigating whether drugs acting on specific genes can improve survival in this patient population. Changes in the immune microenvironment of AML tumors can exacerbate the disease. Immunotherapy has been shown to activate the activity of antileukemic cells that show potential in the treatment of AML^14^. Therefore, it is important to systematically analyze the characteristics of AML tumor immune microenvironment and uncover targets for AML immunotherapy in clinical applications^15^. To diagnose and treat AML patients, it is necessary to understand the survival status of the patients. Prediction and evaluation of clinical prognosis can be facilitated by the discovery of new molecular biomarkers. Hence, the identification of AML prognostic markers having identifiable characteristics, and establishment of AML risk prediction models, will play important roles in the treatment and prognosis of AML patients.

Epigenetic regulations, such as RNA methylation, affect the tumorigenesis and progression of AML^16^. Among these epigenetic regulations, *N6*-methyladenosine (m^6^A), the most common RNA modification in eukaryotic cells, is controlled by a reversible reaction catalyzed by methyltransferases (writers), demethylases (erasers), and methylation-recognition proteins (readers), which regulate m^6^A modifications in RNA^17^. m^6^A methylation also affects RNA epigenetic functions such as mRNA stability, nuclear speed, translation, and degradation^18,19^. Many studies have shown that m^6^A RNA methylation plays an important role in maintaining the balance of self-renewal and differentiation of hematopoietic stem cells (HSCs). For example, the m^6^A reader YTHDF2 suppresses proinflammatory pathways and sustains the function of HSC^20^. m^6^A RNA methylation balances the cellular fate of HSCs by affecting symmetric differentiation^21^. m^6^A methyltransferase METL3 can cause the accumulation of HSCs in the bone marrow and hinder the differentiation of HSCs^22^. Compared with normal HSCs, m^6^A methylation promotes the formation of more phase-separated nuclear bodies in AML cells to maintain the undifferentiated state of leukemia^23^. At present, Studies on m^6^A in AML have been mainly focused on mRNA modifications. For example, METTL 3 and METTL 14 are up-regulated in all subtypes of AML, and overexpression of these two genes promotes the proliferation of AML cells^24,25^. The RNA demethylase, fat mass and obesity-associated protein (FTO), reduces aerobic glycolysis in leukemia cells^26^. Inhibition of m^6^A reader protein YTHDF2 expression promotes apoptosis of leukemic stem cells^27^. The RNA-binding protein YBX1 maintains the survival of myeloid leukemia cells in an m^6^A-dependent manner^28^. These findings indicate that m^6^A modification is closely related to the tumorigenesis and progression of AML. Normal hematopoiesis depends on HSCs, m^6^A RNA methylation also affects the function of HSCs. METTL3 inhibits myeloid differentiation by further activating the translation of related proteins by m^6^A modification on mRNA of genes such as MYC^24^. METTL 14 enhances the mRNA stability and translation of the oncogene transcription factors MYB and MYC^25^. Many studies have shown that YTHDF 2 also plays an important role in maintaining the resting state of HSCs^29,30^. These findings indicate the importance of some m^6^A regulatory factors in normal hematopoietic function.

m^6^A modification also occurs in microRNAs, long non-coding RNAs (lncRNAs), and circular RNAs^31–33^. LncRNAs, which are RNA molecules having a transcript length of more than 200 nucleotides, account for a quarter of the total number of genes in the human genome^34^. lncRNAs may not encode proteins, but do participate in the regulation of coding genes at various levels, thereby playing important roles in various physiological functions of the human body^35^. LncRNAs are also involved in the many biological processes of AML such as in p53- and BCL-2-dependent regulation of AML tumorigenesis^36,37^. Aberrations in lncRNAs abnormally promote self-renewal in HSCs^38^, participate in the epigenetic regulation of chromosomal translation^39^, and regulate glucose metabolism to further regulate the progression of AML^40^. These findings indicate that abnormal expression or regulation of lncRNAs plays an important role in AML tumorigenesis and progression. Many studies have confirmed the existence of a relationship between lncRNAs and m^6^A modification^41^. This relationship has also been shown to affect carcinogenesis^42^. LncRNAs modify the expression of cyclin genes via m^6^A modification and cell-cycle arrest^43^. LncRNAs also promote the phosphorylation and degradation of oncogenes to inhibit the progress of colorectal cancer and are negatively regulated by m^6^A-reading proteins^44^. m^6^A-reading proteins mediate the degradation of lncRNAs and promote the proliferation of endometrial cancer^45^. However, m^6^A-modified lncRNAs, which exert regulatory effects in many diseases, have rarely been examined in AML.

Studying the functions of m^6^A-related lncRNAs in AML may have practical significance for the clinical diagnosis and treatment of patients with AML. In our present study, we used Pearson correlation analysis to identify lncRNAs related to m^6^A regulatory genes, namely m^6^A-related lncRNAs, examined the biological relationship between m^6^A-related lncRNAs and AML, and evaluated the role of lncRNAs in prognostic prediction. We also extracted the expression profiles of lncRNAs and m^6^A regulatory genes from the Cancer Genome Atlas (TCGA) database, analyzed the characteristics of m^6^A-related lncRNAs, and examined their roles in the AML tumor immune microenvironment. Additionally, we established and verified a new prognostic model based on m^6^A-related lncRNAs for the prediction of overall survival (OS) in patients with AML.

## Materials and methods

### Data collection and processing

RNA-sequencing (RNA-seq) data for the blood samples, and corresponding clinical information on 151 AML patients and 755 healthy participants, were downloaded from The Cancer Genome Atlas (TCGA, https://portal.gdc.cancer.gov/) and Genotype-Tissue Expression (GTEx, https://www.gtexportal.org/) databases, two gene expression profile data sets (gse65263 and gse114868^46^) were obtained from gene expression compilation (GEO, https://www.ncbi.nlm.nih.gov/geo/) database, respectively, normalized gene expression was measured as fragments per kilobase of transcript per million mapped reads (FPKM) and log2-based transformation, and the two RNA-seq datasets were then combined. The R x64 4.0.3 software package was used for data analysis. Using human genome annotation data, we identified the lncRNAs in the dataset, and extracted data on the expression of 23 m^6^A regulatory genes (i.e., *METTL3*, *METTL14*, *METTL16*, *WTAP*, *VIRMA*, *ZC3H13*, *RBM15*, *RBM15B*, *YTHDC1*, *YTHDC2*, *YTHDF1*, *YTHDF2*, *YTHDF3*, *HNRNPC*, *FMR1*, *LRPPRC*, *HNRNPA2B1*, *IGFBP1*, *IGFBP2*, *IGFBP3*, *RBMX*, *FTO*, and *ALKBH5*) that had been described extensively in previous studies. The ggpubr package was used to construct a box plot showing differential gene expression. Next, we used the limma package to screen out m^6^A-related lncRNAs based on analysis using Pearson’s correlation coefficient (absolution correlation coefficient > 0.6, P < 0.001). Then, we used igraph to map the co-expression networks of m^6^A regulatory genes and related lncRNAs.

### Identification and expression analysis of m^6^A-related lncRNAs associated with AML prognosis

The RNA-seq data obtained for AML patient samples were merged with the corresponding patient clinical information, followed by removal of samples for which information pertaining to survival time or status was unavailable. The “survival” package was used to perform univariate Cox regression analysis to identify the m^6^A-related lncRNAs associated with AML prognosis at the significance cutoff of P < 0.001. Expression of m^6^A-related lncRNAs associated with AML prognosis was evaluated in samples obtained from AML patients and healthy participants. ggpubr and pheatmap packages were used to construct the box plots and heatmap.

### Relationship between m^6^A-related lncRNAs clustering subgroups and AML clinicopathological characteristics evaluated using consensus cluster analysis

Next, we investigated the relationship between m^6^A-related lncRNAs and clinicopathological characteristics of AML. The ConsensusClusterPlus package (50 iterations, sampling rate of 80%) was used to perform a consensus cluster analysis on AML samples based on the expression of m^6^A-related lncRNAs associated with AML prognosis. Then, different AML samples were divided into different subgroups, and the biological characteristics of the subgroups were analyzed to evaluate the relationship between m^6^A-related lncRNAs and AML. Subsequently, the “survival” package was used to analyze the survival of different subgroups. The pheatmap package was used to visualize the expression of m^6^A-related lncRNAs associated with AML prognosis, and to describe the characteristics of clinicopathological factors among the different subgroups. The ggplot2 and ggpubr packages were used to compare the immune checkpoint, expression of programmed death-ligand 1 (PD-L1), between the subgroups, and between tumor-bearing and healthy controls. Lastly, the corrplot package was used to analyze correlations between PD-L1 expression and 15 m^6^A-related lncRNAs associated with AML prognosis.

### Gene set enrichment analysis (GSEA) and evaluation of AML tumor immune microenvironment, and Analysis of the relationship between the clustering subtypes of m^6^A-related lncRNAs and the biological characteristics of AML

GSEA was used to analyze the relationship between the m^6^A-related lncRNAs clustering subtypes and biological characteristics of AML. GSEA was also used to compare the abnormally activated Kyoto Encyclopedia of Genes and Genomes (KEGG) signaling pathways between the differently clustered subgroups^47^. The CIBERSORT algorithm was used to calculate the infiltration-ratio scores for 22 types of immune cells in the clustering subgroups based on the expression of characterizing genes. The sum of the infiltration ratio scores for each immune cell was 1. The ESTIMATE algorithm was used to calculate the ratio of cell components, including immune and stromal cells, in the clustering subgroups, indirectly reflecting the purity of tumors between the different subgroups.

### Screening of risk-related signals, and risk-model construction and verification

Cox regression analysis with least absolute shrinkage and selection operator (LASSO) penalty was used to identify the combination of m^6^A-related lncRNAs having the highest prognostic value. Optimal model parameters were used for model construction. The TCGA AML queen was randomly divided into a training set and a test set. The risk score of AML patients in the training set was calculated using the following equation:

Risk score = AFF2-IT1 × (− 0.318973988531144) + LINC02593 × (− 0.0297508127431019) + AC000120.2 × (− 0.0972888222752296) + AC048382.1 × (− 1.04822722017167) + AL391834.1 × (− 0.0598646684481577) + TRAF3IP2-AS1 × (− 0.266546979989193), where gene ID refers to the expression level of each gene, and each value after the gene ID refers to the coefficient of that gene. AML patients were divided into a low-risk group and a high-risk group using median risk score. The “survival” package was used to analyze the survival of patients within the two groups, and the pheatmap package was used to construct heatmaps of model factors, patient survival time, and survival curves of the two groups. Receiver operating characteristics (ROC) curve analysis was used to evaluate the accuracy of the risk prediction model.

### Independent prognostic analyses of the risk model

Next, we used univariate and multivariate independent prognostic analyses of risk scores and clinicopathological factors (i.e., gender, age, and FAB classification) to evaluate the independent prognostic value of the risk prediction model. Factors with a P < 0.05 were considered prognostic-related factors. In addition, all AML patients were stratified according to their age and gender into an elderly group (≥ 60 years old) and a young group (< 60 years old), and into a male group and female group, to calculate the risk score for all the patients in different groups. Patients were then divided into high- and low-risk groups based on median risk score, and were evaluated using survival analysis to determine the survival status of patients in different groups. The independent prognostic values of the risk prediction model were then further evaluated based on the prognostic analyses described above.

### Correlation analysis of risk-related signaling, clinical characteristics of AML, and immune-cell infiltration

The ggpubr package was used to analyze the distribution of risk scores with respect to different clinical characteristics (i.e., clinical pathologic factors, subgroup clustering of m^6^A-related prognostic lncRNAs, and immune-cell ratio scores). The ggplot2, ggpubr, and ggExtra packages were used to analyze correlations between the level of immune-cell infiltration and risk scores. P < 0.05 was considered statistically significant.

### In vitro assays

This work was approved by the Ethics Committee of the Second Affiliated Hospital of Nanchang University. Informed consent was obtained from all participants. All methods were performed in accordance with the relevant guidelines and regulations. We collected peripheral blood samples of newly diagnosed AML patients and extracted mononuclear cells. The monocyte THP1 cell line was cultured in RPMI1640 medium containing 10% fetal bovine serum and 1% penicillin–streptomycin and incubated in a humidified atmosphere incubator at 37 °C and 5% CO_2_. A lentivirus containing the SRSF10 siRNA was purchased from Hanbio (Shanghai, China). THP1 cells were infected with the lentivirus and selected for puromycin resistance. After RNA extraction and reverse transcription, a TAKARA kit (Japan) was used to perform real-time polymerase chain reaction (RT-PCR) on the ABI7500 instrument to determine the level of gene expression in THP1 cells and peripheral blood mononuclear cells from the patients. Western blot analysis was used to assess the knockdown efficiency of SRSF10 in THP1 cells. Antibodies used were rabbit anti-β-tubulin (1:10,000, #2146) and anti-SRSF10 (1:1000, 42267S) from Cell Signaling Technology (Danvers, MA, USA). The sequences of primes and siRNA are shown in Supplementary Table 1.

## Results

### Expression of m^6^A-regulatory genes in AML, and identification of related lncRNAs

**Figure 1:**
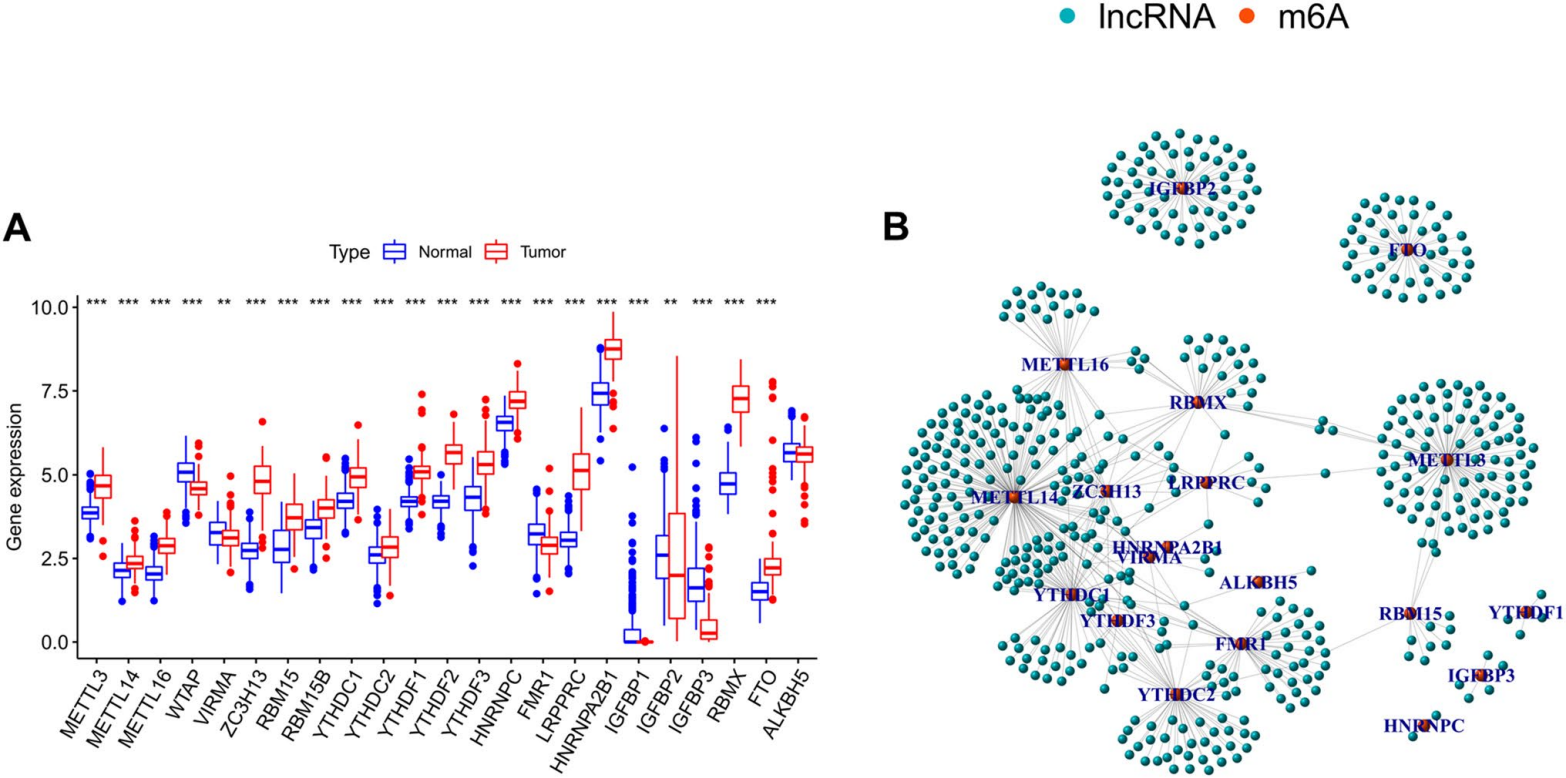
Identifying m^6^A regulatory genes and related lncRNAs. (**A**) The levels of expression of m^6^A regulatory genes in tumor and normal samples. The Wilcoxon test was used to determine the statistical significance of the difference, *P < 0.05, **P < 0.01, ***P < 0.001. (**B**) Correlation network of m^6^A regulatory genes and related lncRNAs in AML, blue nodes are m^6^A-regulated lncRNAs whose expression correlate with the m^6^A regulators, and that lines indicate correlations.

The m^6^A regulatory genes are important regulators in tumorigenesis and progression of AML. In our present study, we performed a differential expression analysis using AML and normal samples. Compared with that of normal samples, AML samples showed significantly higher expression of METTL3, METL14, METTL16, ZC3H13, RBM15, RBM15B, YTHDC1, YTHDC2 YTHDF1, YTHDF2, YTHDF3, HNRNPC, LRPPRC, HNRNPA2B1, RBMX, and FTO. the expression of WTAP, VIRMA, FMR1, IGFBP1, IGFBP2, and IGFBP3 in normal samples was significantly higher than that in AML samples (P < 0.05) (Fig. 1A). We then extracted the expression profiles of 14,086 lncRNAs and 23 m^6^A regulatory genes from AML samples obtained from the TCGA database. Using correlation coefficient to evaluate the relationship between m^6^A regulatory genes and lncRNAs, we show that there were 525 m^6^A-regulated lncRNAs and 680 interactions (Fig. 1B, Supplementary Table 2) (absolute correlation coefficient > 0.5, P < 0.001).

### Prognostic analyses of m^6^A-related lncRNAs

**Figure 2:**
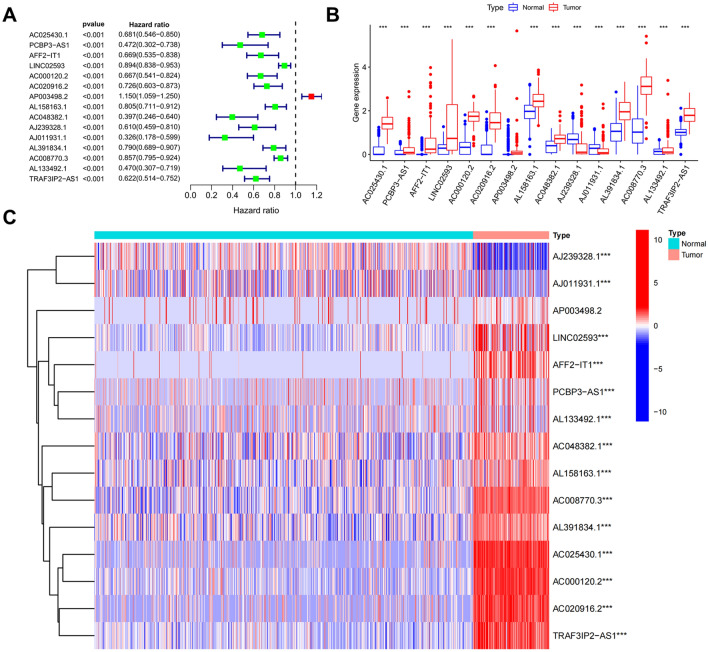
Prognostic analyses of m^6^A-related lncRNAs and expression of related factors. (**A**) Forest plot, showing 15 m^6^A-related lncRNAs associated with the overall survival rate of AML patients, constructed based on univariate Cox regression analysis. (**B**,**C**) Box plot and heatmap showing the overall and single-sample expression of m^6^A-related prognostic lncRNAs, respectively, in tumor and normal samples. *P < 0.05, **P < 0.01, ***P < 0.001.

Using univariate Cox proportional hazard regression analysis, 15 lncRNAs, shown significant in AML prognosis, were screened out from the abovementioned 525 m^6^A-regulated lncRNAs (P < 0.001, Fig. 2A). Among these 15 lncRNAs, AP003498.2 was a survival risk factor in AML patients, while the remaining lncRNAs were protection factors. We then analyzed the expression levels of these m^6^A-related prognostic lncRNAs. Our results show that the expression levels of AC025430.1, AFF2-IT1, LINC02593, AC000120.2, AL158163.1, AC048382.1, AL391834, AC008770.3, and AL133492.1 in AML samples were significantly higher than those in normal samples. The expression levels of AC020916.2 and AJ239328.1 in AML samples were significantly lower than those in normal samples (Fig. 2B). Heatmap shows single-sample expression of m^6^A-related prognostic lncRNAs in AML and normal-tissue samples (Fig. 2C).

### m^6^A-related lncRNAs clustering subgroups are associated with clinicopathologic characteristics of AML

**Figure 3:**
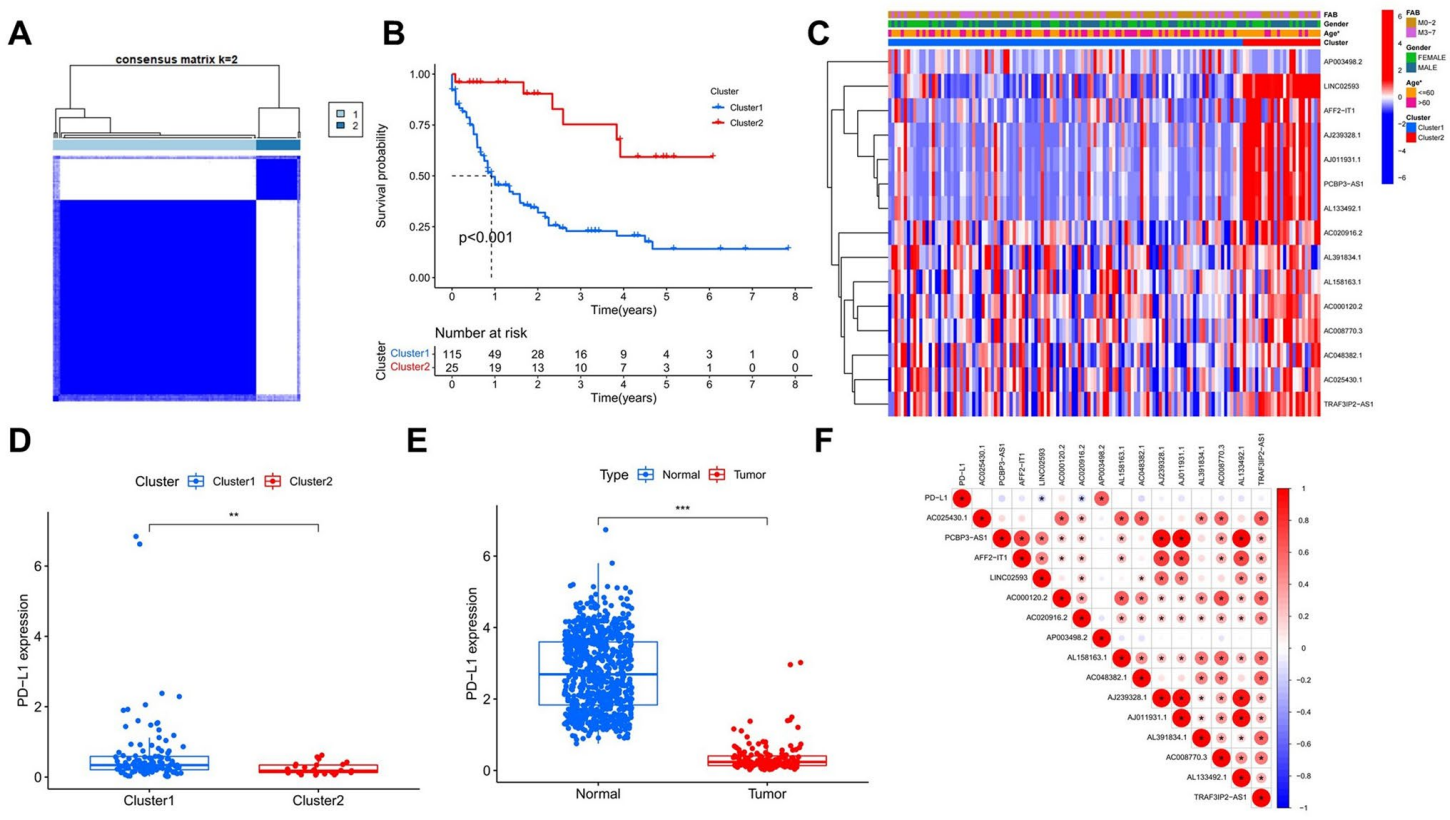
Analysis of clinicopathologic characteristics of AML in cluster subgroups. (**A**) Cluster discrimination was highest for consensus clustering matrix of k = 2. (**B**) Kaplan–Meier survival analysis of the subgroups of AML patients in cluster1 and cluster2. (**C**) clinicopathologic characteristics and m^6^A-related lncRNA expression associated with AML prognosis between the two clusters. (**D**) PD-L1 expression level in cluster1 and cluster2. (**E**) PD-L1 expression level in AML samples and normal samples. (**F**) Correlation analysis of m^6^A-related lncRNAs associated with AML prognosis and PD-L1. The size of the dot represents the correlation coefficient, and the larger the dot, the higher the correlation. *P < 0.05, **P < 0.01, ***P < 0.001.

To explore the relationship between m^6^A-related lncRNAs and clinicopathological characteristics of AML, we used unsupervised cluster analysis to analyze 151 AML samples based on TCGA RNA-seq data of 15 m^6^A-related prognostic lncRNAs. The 151 AML samples were divided into two clusters having the highest stabilities (Fig. 3A). First, we evaluated the two clusters using survival analysis. Our results indicate that patients in cluster1 showed significantly worse OS that that of patients in cluster2 (P < 0.001, Fig. 3B). Then, we investigated whether there were differences in clinicopathologic factors (i.e., gender, age, and FAB classification), as well as in the expression of the immune checkpoint molecule PD-L1, between the two clusters, and analyzed correlations between the expression levels of PD-L1 and m^6^A-related lncRNAs. Cluster2 had more elderly patients, and the expression levels of m^6^A-related prognostic lncRNAs were generally higher in Cluster2 than in Cluster1 (Fig. 3C). PD-L1 showed differential expression in the two clusters, as well as in AML and normal samples (Fig. 3D,E). Gene correlation analysis shows that PD-L1 expression was negatively correlated with LINC02593 and AC020916.2 co-expression, and positively correlated with AP003498.2 co-expression (Fig. 3F). AP003498.2 was shown a high-risk lncRNA in AML prognosis. High expression of AP003498.2 was associated with increased risk of death in patients with AML. High expression of PD-L1 may promote the immune escape of AML cells. These results indicate that there are differences in clinicopathological characteristics between the clustering subgroups of m^6^A-related lncRNAs, which were related to the age and survival of AML patients. PD-L1 also has differences in expression between the two subgroups.

### m^6^A-related lncRNAs clustering subgroups are associated with biological characteristics of AML

**Figure 4:**
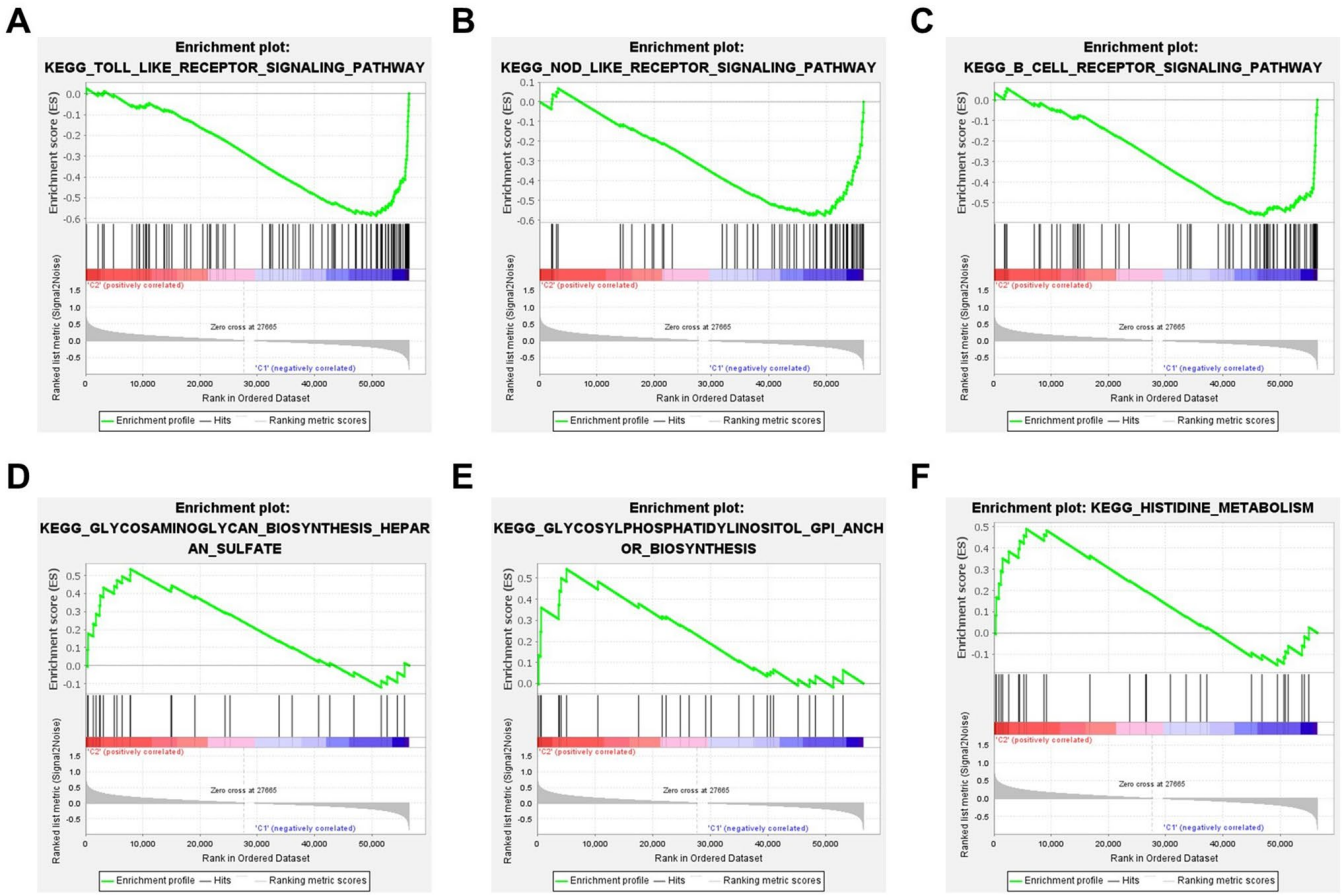
Abnormally activated signaling pathways in the two subgroups after gene-set—enrichment analysis. (**A**–**C**) Performed in cluster1. Including Toll-like receptor signaling pathway (ES = 0.59, P = 0.002, FDR = 0.042), NOD-like receptor signaling pathway (ES = 0.58, P = 0.006, FDR = 0.046), B-cell receptor signaling pathway (ES = 0.56, P = 0.006, FDR = 0.044). (**D**–**F**) performed in cluster2. Including glycosaminoglycan biosynthesis heparan sulfate (ES = 0.54, P = 0.007, FDR = 0.039), glycosylphosphatidylinositol GPI anchor biosynthesis (ES = 0.54, P = 0.003, FDR = 0.042), histidine metabolism (ES = 0.49, P = 0.005, FDR = 0.041).

Next, we analyzed differences in the biological responses of the subgroups generated using consensus clustering in order to further explore the relationship between m^6^A-related lncRNAs clustering subgroups and AML biological characteristics. GSEA was used to explore the main KEGG signaling pathways in the two subgroups. Our results show that cluster1 was mainly involved in Toll-like receptor signaling, NOD-like receptor signaling, and B-cell receptor signaling pathways (Fig. 4A–C), which are related to immunomodulation. Cluster2 was mainly involved in the metabolism of various substances, such as histidine metabolism, and heparan sulfate and glycosylphosphatidylinositol (GPI) anchored protein biosynthesis (Fig. 4D–F), which are important for adhesion, proliferation, invasion, and metastasis of cancer cells. These results indicate that there are differences in biological characteristics between clusters, which affected tumorigenesis and progression of AML, and survival of AML patients.

### Immune microenvironment is different between the clustering subgroups

**Figure 5:**
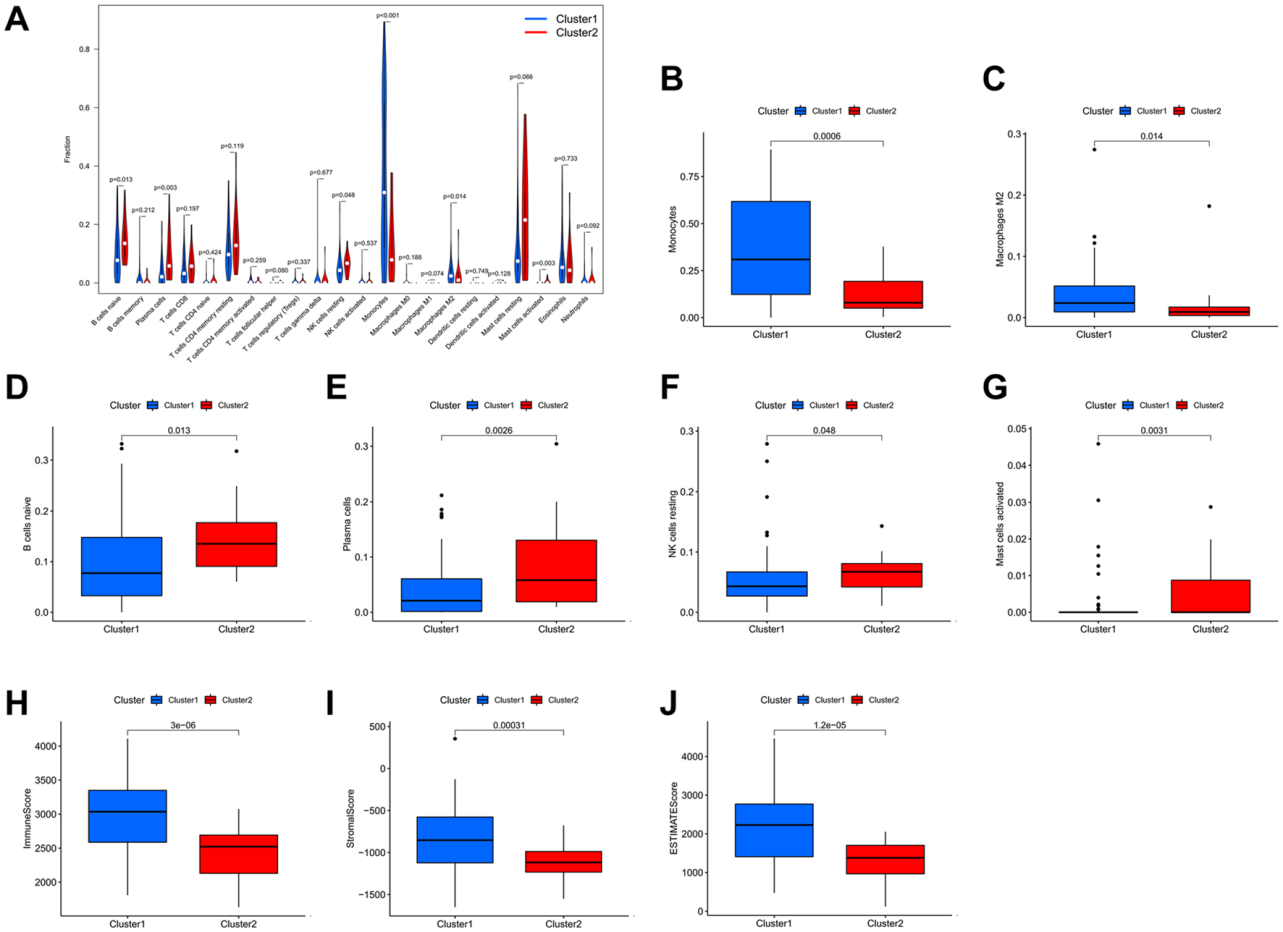
Characteristics of AML tumor immune microenvironment in different cluster subgroups. (**A**) A violin plot showing the ratios of 22 immune cells among clustered subgroups. (**B**–**G**) immune cells with different levels of expression in clustered subgroups. (**H**–**J**) Differences in immune, stromal, and ESTIMATE scores in different clustered subgroups.

To evaluate the characteristics of tumor immune microenvironment in the two clusters, we analyzed the levels of immune cell infiltration and tumor purity in the clusters. The CIBERSORT algorithm was used to calculate infiltration ratio scores for 22 different immune cells in 151 AML samples. The median scores for different types of immune cells in the two clusters were analyzed and compared (Fig. 5A). The ESTIMATE algorithm was used to score the ratio of immune and stromal cells in the tumor microenvironment of patients in cluster1 and cluster2. Our results indicate that infiltration levels of monocytes and M2 macrophages in cluster1 were significantly higher than those in cluster2 (Fig. 5B,C). The infiltration levels of naïve B cells, plasma cells, resting natural killer (NK) cells, and activated mast cells were significantly higher in cluster2 than in cluster1 (Fig. 5D–G). In addition, the immune score, stromal score, and ESTIMATE score of cluster1 were higher than those of cluster2 (Fig. 5H–J). These results indicate that the levels of immune cell infiltration differed considerably between the two groups.

### Risk signaling of m^6^A-related lncRNAs and risk prediction model show prognostic value in AML

**Figure 6:**
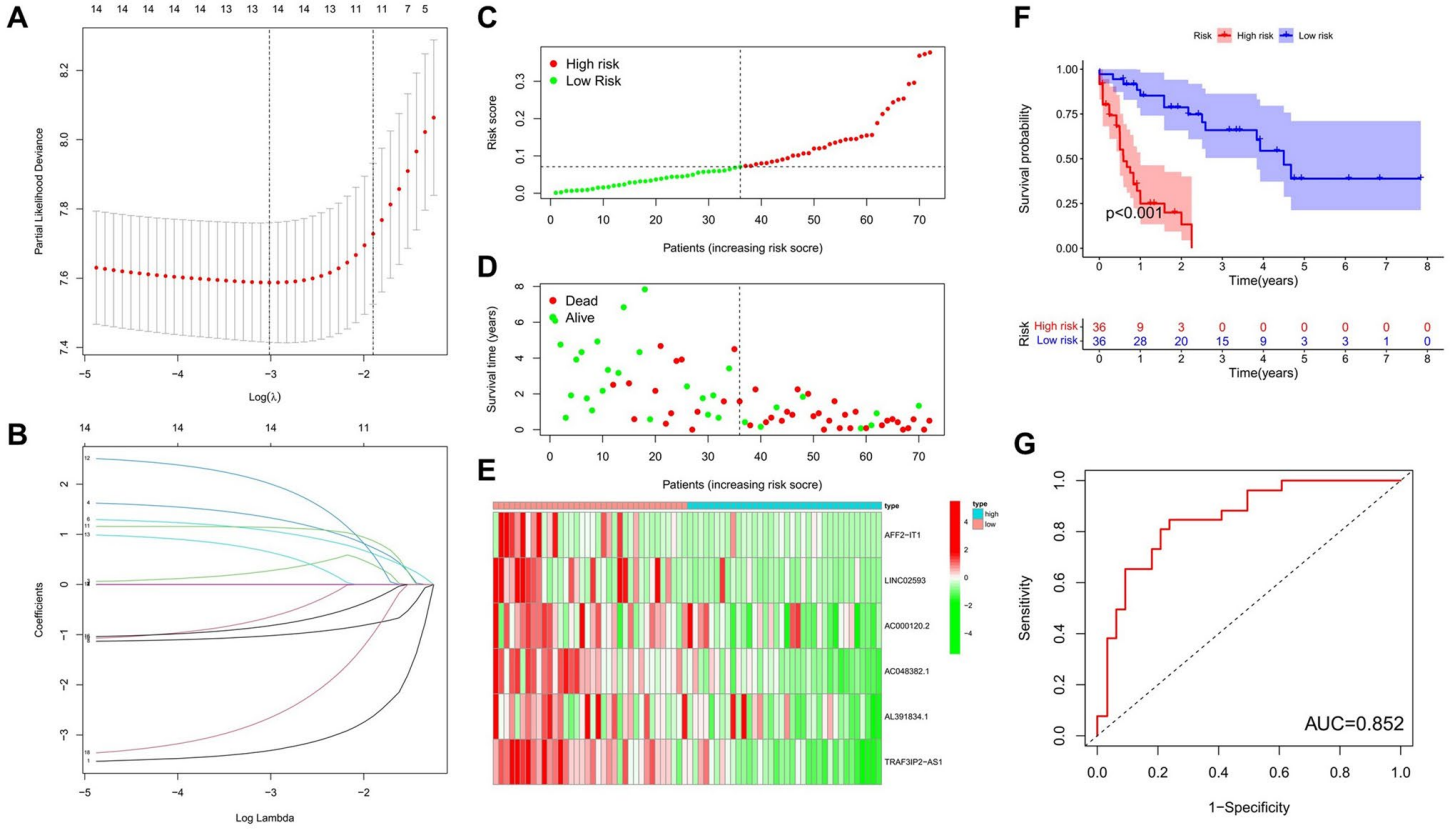
Risk prediction model for AML patients constructed based on risk-associated signaling of m^6^A-related lncRNAs, and analysis of prognostic values in training set. (A) Shows the Log Lambda value corresponding to the minimum cross-validation error point. (**B**) The coefficient of m^6^A-related lncRNAs varies with the Log Lambda value, and the m^6^A-related lncRNAs with non-zero coefficient corresponding to the same Log Lambda value in figure A were selected for subsequent model construction. (**C**) Risk score distribution of the training set based on m^6^A-related lncRNA risk prediction model. (**D**) Survival time and status in the high-risk and low-risk groups in the training set. (**E**) Heatmap showing the expression levels of six m^6^A-related lncRNAs in the model of each patient from the high-risk and low-risk groups in the training set. (**F**) Kaplan–Meier curve analysis of OS in the high-risk group and low-risk group in the training set. (**G**) The ROC curve for predicting the prognoses of patients in the training set.

**Figure 7:**
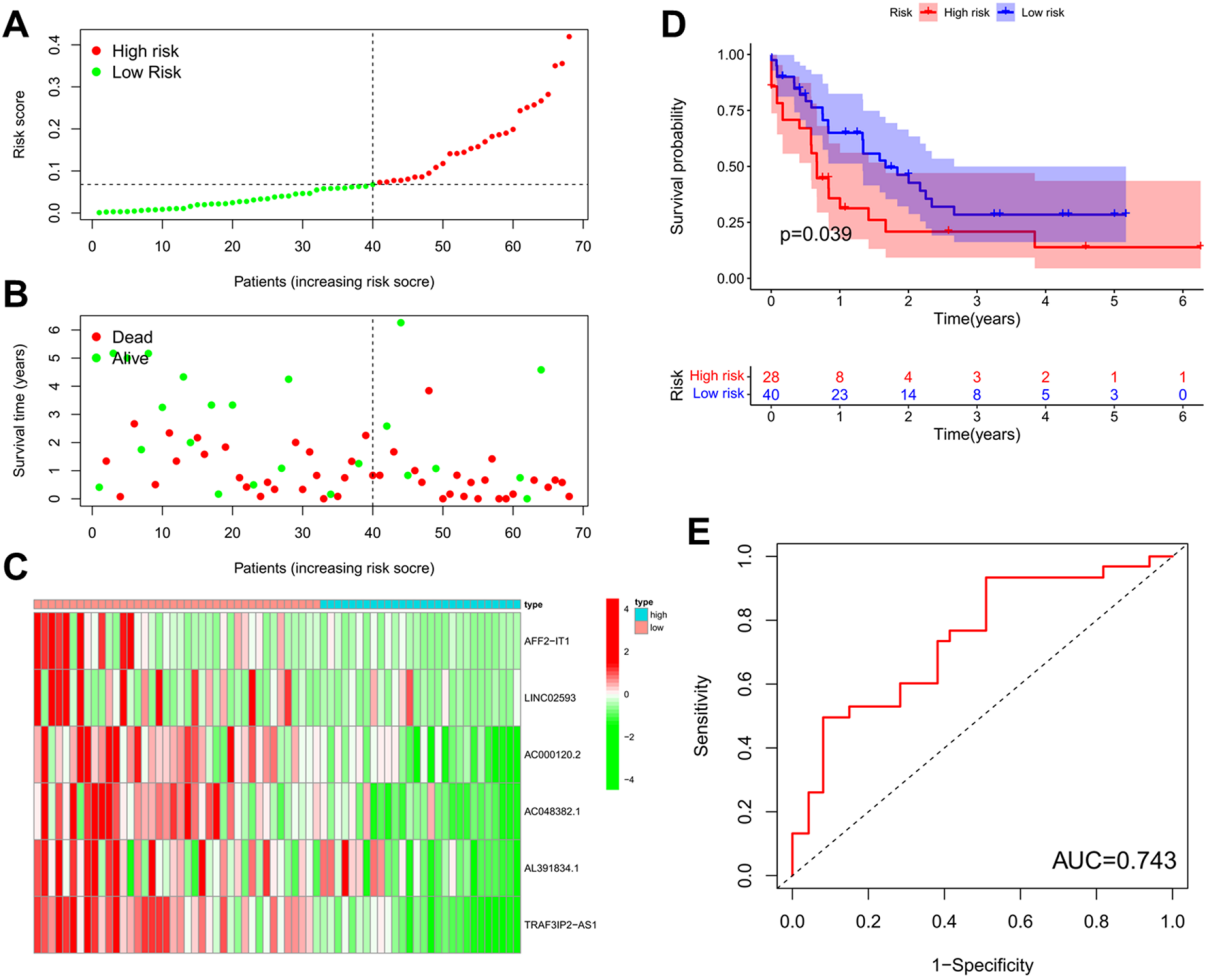
Prognostic performance of risk prediction model in the test set. (**A**) Distribution of risk scores obtained using m^6^A-related lncRNA model in the test set. (**B**) Comparison of survival time and status in the high-risk and low-risk group in the test set. (**C**) Heatmap showing the expression levels of six m^6^A-related lncRNAs in the model of each patient from the high-risk and low-risk groups in the test set. (**D**) Kaplan–Meier curve analysis of OS in the high-risk group and low-risk group in the test set. (**E**) The ROC curve for predicting the prognoses of patients in the test set.

Using LASSO regression analysis, we screened out the six most representative combinations of m^6^A-related lncRNAs from the 15 m^6^A-related prognostic lncRNAs, and established a risk prediction model to evaluate the prognostic value of m^6^A-related lncRNAs in AML (Fig. 6A,B). To ensure the accuracy of the model, 140 AML patients were randomly assigned to a training set (n = 72) and a test set (n = 68) for the construction and verification of the model. After calculating the risk scores of individual patients in the training set, patient samples were divided into a high-risk group and a low-risk group based on median risk score (Fig. 6C). Survival curves show that the number of patient deaths increased as the risk score increased, indicating that the risk score was related to the survival status of AML patients (Fig. 6D). Heatmap shows changes in the risk score and expression levels of lncRNAs. The expression levels of lncRNAs used in the construction of the risk prediction model in the high-risk group were generally lower than those in the low-risk group (Fig. 6E). Analysis of patient survival indicates that the OS of the low-risk group was higher than that of the high-risk group (P < 0.001, Fig. 6F). Area under the curve (AUC) of the ROC curve was 0.852, indicating that the model showed high accuracy in predicting the prognosis of AML patients (Fig. 6G). Next, we used the model to calculate risk scores for individual patients in the test set in order to verify the risk prediction model established using the training set. Our results show that the characteristics of the test set were consistent with those of the training set (Fig. 7A–E), indicating that m^6^A-related lncRNAs had prognostic value, and that the risk prediction model showed satisfactory performance in predicting the prognosis of patients with AML.

### Independent predictive effect of AML prognosis in the risk prediction model

**Figure 8:**
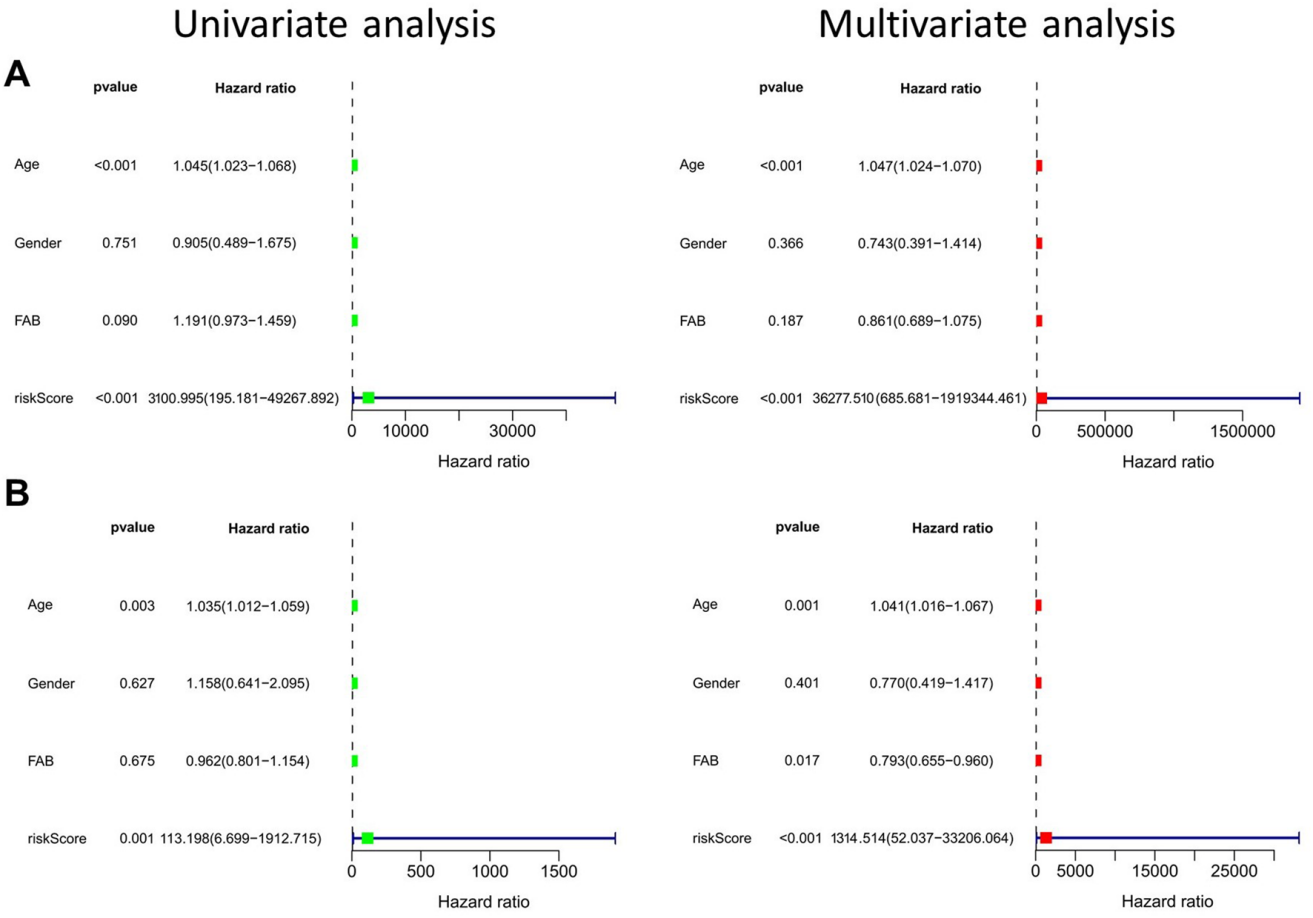
Identification of independent prognostic factors in AML cohort. Univariate and multivariate independent prognostic analyses of clinicopathologic factors and risk scores were conducted using the training set (**A**) and the test set (**B**).

**Figure 9:**
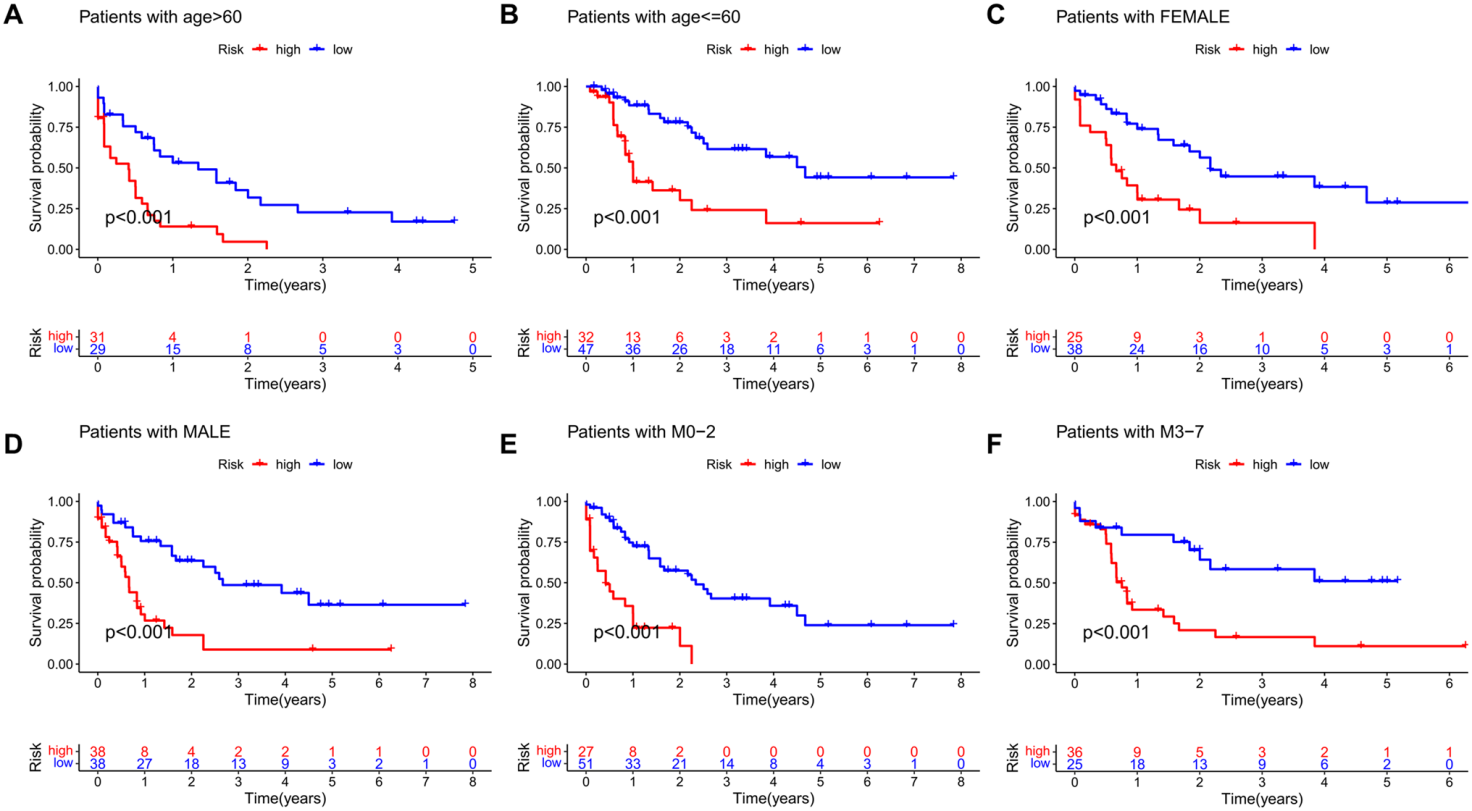
Survival analysis of cohorts stratified by clinicopathological factors. Kaplan–Meier survival curves showing differences in OS between the high-risk and low-risk groups in the entire TCGA AML cohort stratified by age (**A**,**B**), gender (**C**,**D**) and FAB classification (**E**,**F**).

To further verify whether the risk prediction model was applicable in patients with varying clinicopathological factors, we used the univariate and multivariate independent prognosis analyses in the training and test sets. Both analyses show that risk score was a prognostic factor independent of clinicopathologic factors (P < 0.001, Fig. 8A,B). Subsequently, patients were stratified according to their clinical pathologic factors including gender, age, and FAB classification, and their risk scores were calculated. Median score was used to distinguish the high-risk and low-risk groups. Survival analysis shows that in each stratified subgroup, patients in the low-risk group showed higher OS than that of patients in the high-risk group (Fig. 9A–F). These results indicate that the m^6^A-related lncRNA risk prediction model could predict the prognosis of AML patients without being confounded by gender, age, and FAB classification.

### Risk score is correlated with clinicopathological factors and immune infiltration levels

**Figure 10:**
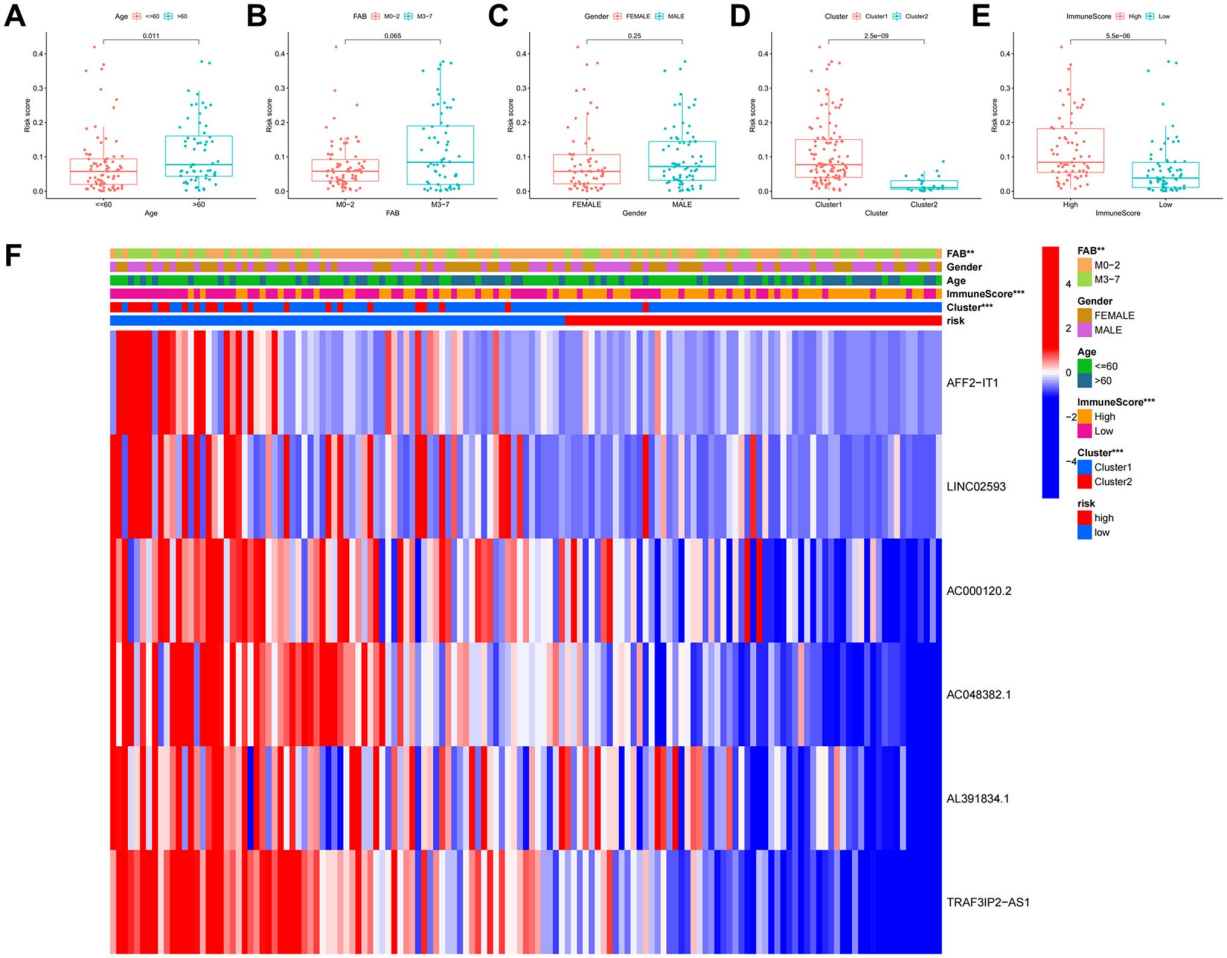
Correlation between risk scores, clinicopathologic factors, and immune infiltration in AML patients. (**A**–**E**) Distribution of risk scores with respect to age, FAB classification, gender, consensus clustering subgroups of m^6^A-related lncRNAs associated with AML prognosis, and immune score grouping. (**F**) Heatmap showing the distribution of patients grouped by age, gender, consensus clustering of m^6^A-related lncRNAs associated with AML prognosis, and immune scores between the high-risk and low-risk AML group. *P < 0.05, **P < 0.01, ***P < 0.001.

**Figure 11:**
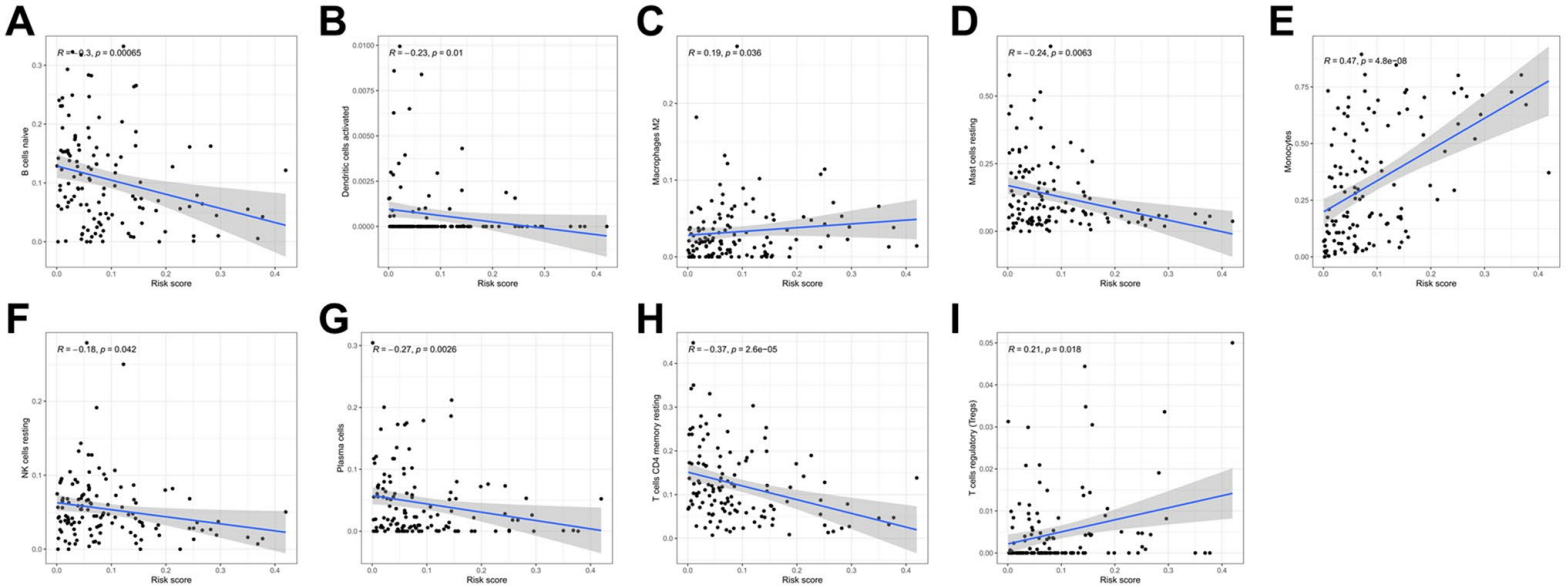
Correlation between risk scores and levels of immune cell infiltration in AML. Notes: immune cells included naïve B cells (**A**), activated dendritic cells (**B**), M2 macrophages (**C**), resting mast cells (**D**), monocytes (**E**), resting NK cells (**F**), plasma cells (**G**), resting memory T cells CD4 (**H**), and regulatory T cells (Tregs) (**I**).

To evaluate the characteristics of risk signaling in m^6^A-related lncRNA, we analyzed the relationship between risk scores, clinicopathological factors, and immune infiltration levels. Patients were grouped according to their age, gender, clustering subgroups of m^6^A-related prognostic lncRNAs, and scores obtained for the immune cell ratio, in order to study risk correlation with respect to the grouping of each clinical factor. Our results show that risk scores in the elderly group (≥ 60 years old) were significantly increased compared with those of the young group (< 60 years old, P < 0.05, Fig. 10A). No significant difference in risk scores was found between the genders and between the FAB classifications (Fig. 10B,C). In the clustering subgroups of m^6^A-related prognostic lncRNAs, the risk score of cluster1 was significantly higher than that of cluster2 (P < 0.001, Fig. 10D). Comparison of groups with high and low immune scores, categorized based on median immune score, showed that the risk score of the group with high immune scores was significantly higher than that of the group with low immune scores (P < 0.001, Fig. 10E). Heatmap confirmed the above results, and shows that the expression levels of the six lncRNAs used in model construction were low in the high-risk group (Fig. 10F). Further analysis of the relationship between various types of immune cells and risk scores shows that risk scores were significantly related to naïve B cells (R = − 0.3, P = 0.00065), activated dendritic cells (R = − 0.23, P = 0.01), M2 macrophages (R = 0.19, P = 0.036), resting mast cells (R = − 0.24, P = 0.0063), monocytes (R = 0.47, P = 4.8e−08), resting NK cells (R = − 0.18, P = 0.042), plasma cells (R = − 0.27, P = 0.0026), resting memory CD4 T cells (R = − 0.37, P = 2.6e−05), and regulatory T cells (Tregs) (R = 0.21, P = 0.018), and were positively correlated with M2 macrophages, monocytes, and Tregs (Fig. 11A–I).

### Interaction between SRSF10 and TRAF3IP2

**Figure 12:**
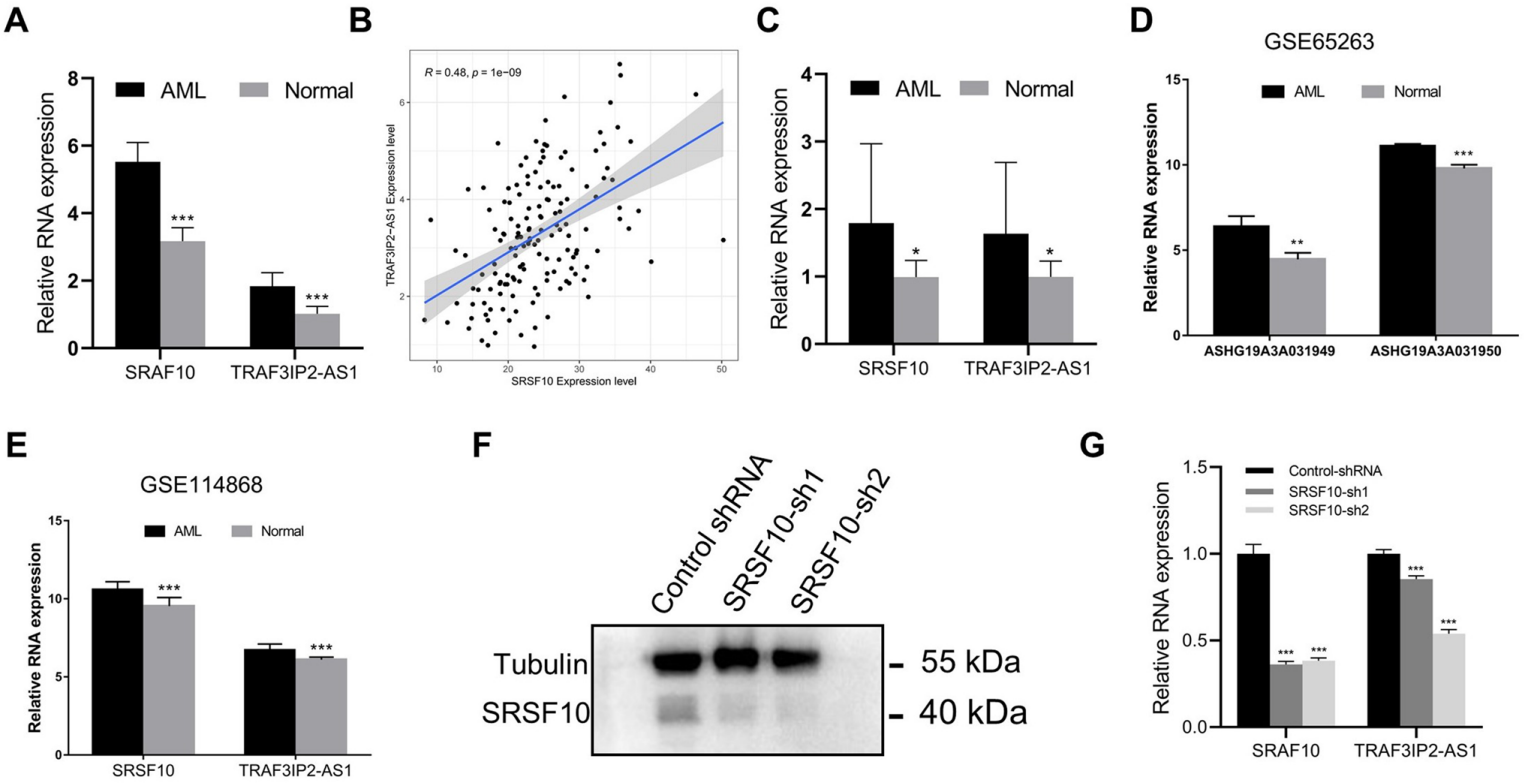
Interaction between SRSF10 and TRAF3IP2. (**A**) The levels of expression of SRSF10 and TRAF3IP2-AS1 in AML samples from the TCGA databases and normal samples from the GTEx databases. (**B**) Correlation analysis between SRSF10 and TRAF3IP2-AS1 in AML samples from the TCGA database. (**C**) The expression levels of SRSF10 and TRAF3IP2-AS1 in AML peripheral blood samples (n = 15) and normal human peripheral blood samples (n = 15). (**D**,**E**) SRSF10 and TRAF3IP2-AS1 expression levels in AML patient samples and normal samples in GEO chip data (ASHG19A3A031949: TRAF3IP2-AS1 transcript variants 1 and 3, ASHG19A3A031949: TRAF3IP2-AS1 transcript variants 1 and 2). (**F**) SRSF10 protein expression level in control shRNA and SRSF10-sh THP1 cells. (**G**) SRSF10 and TRAF3IP2-AS1 RNA expression levels in control shRNA and SRSF10-sh THP1 cells.

TRAF3IP2-AS1 was selected as a candidate lncRNA to explore its Combined RBPs. We first made predictions on the website ENCORI (http://starbase.sysu.edu.cn/)^48^. These showed that the SRSF10 protein has binding regions for nine transcripts of TRAF3IP2-AS1 in chr6: 111821214–111821244[+] (GSE71096). Compared with normal samples from GTEx databases, the levels of expression of SRSF10 and TRAF3IP2-AS1 were significantly upregulated in AML samples from TCGA databases (Fig. 12A). The expression level of SRSF10 also positively correlated with TRAF3IP2-AS1 expression in TCGA AML samples (Fig. 12B). Next, we verified that the levels of expression of SRSF10 and TRAF3IP2-AS1 were also upregulated in AML samples by RT-PCR (Fig. 12C), and the GEO chip data (GSE65263, GSE114868) showed a similar trend (Fig. 12D,E). Next, we tested the interaction between SRSF10 and TRAF3IP2-AS1 by siRNA targeting SRSF10 in THP-1 cells (Fig. 12F, the original blots are shown in Supplementary Fig. 1). RT-PCR showed that the expression of TRAF3IP2-AS1 was significantly downregulated after SRSF10 knockdown (Fig. 12G). These results indicated that SRSF10 and TRAF3IP2 have Interaction relationship in AML.

## Discussion

AML has a complicated pathogenesis, and our understanding of its etiology and the conditions involved remains limited. Diagnosis of AML is usually based on the evaluation of morphological features of the tissue, immunophenotyping analysis^12^, and grouping using cytogenetics^49^. Few studies have investigated the use of biomarkers for AML prognosis, highlighting the need to explore these molecules and study their impact on the tumorigenesis and progression of AML. lncRNAs have been shown to affect cell proliferation, apoptosis, and the cell cycle in AML^50–52^. LncRNAs also affect the differentiation of bone marrow hematopoietic cells, and are regarded targets in differentiation-induction therapy^53^. In addition, the regulatory mechanisms involved in lncRNA-mediated regulation of AML indicate that lncRNAs can be used as potential molecular markers to predict disease course and survival status in patients with AML^54^. m^6^A methylation plays key roles in the tumorigenesis and progression of AML^55^. However, few studies have examined the roles of m^6^A methylation and lncRNA co-regulation in AML. Based on previous findings on m^6^A methylation and functions of lncRNAs, we investigated the relationship between m^6^A-related lncRNAs and clinical characteristics of AML, evaluated the characteristics of the AML tumor immune microenvironment, and constructed a risk prediction model to predict the prognosis of patients with AML.

Using a TCGA-AML data set, we identified 525 lncRNAs related to 23 m^6^A regulatory genes. Cox regression analysis identified 15 m^6^A-related lncRNAs that were significantly related to the prognosis of AML patients. Most lncRNAs showed high expression level associated with favorable prognosis. We observed more expression of most of these 15 m^6^A-related lncRNAs in samples from AML patients than in those from normal controls, which indicate that these molecules are protective factors, and their expression may be activated in AML and further inhibit tumor development. There are similar characteristics in many studies^56,57^. To further explore the relationship between m^6^A-related lncRNAs, and the clinicopathologic and biological characteristics of AML, we performed a cluster analysis based on the expression profiles of m^6^A-related prognostic lncRNAs. This analysis yielded two subgroups, which we designated as cluster1 and cluster2. There were significant differences in age and FAB classification between the two clusters. The expression of the m^6^A-related prognostic lncRNAs was upregulated in cluster2 compared with that in cluster1. The OS of patients in cluster2 was also significantly increased compared with that of patients in cluster1. The upregulated expression of these lncRNAs indicate a favorable prognosis in patients with AML. Analysis of the enrichment of KEGG signaling pathways in the two clusters showed significant differences. The pathways enriched in cluster1 were mainly related to immune regulation. Including Toll-like receptor signaling pathway, NOD-like receptor signaling pathway, and B-cell receptor signaling pathway. Increasing evidence suggests that m^6^A methylation and lncRNAs are involved in immune regulation and inflammatory responses^58^. To explore the characteristics of the immune microenvironment between two clusters, we performed an algorithm evaluation analysis. Our results indicate that the degree of immune-cell infiltration and tumor purity in the two subgroups were significantly different. In cluster2, we observed an increased number of cells of the monocyte macrophage lineage, Especially M2 macrophages, which promote tumor progression^59,60^. Studies have shown that the expression of Toll like receptors on the surface of tumour cells in the tumour microenvironment, to varying degrees, contributes to creating an environment that favours the tumour but not the immune effector cells^61^. M2 macrophages promote liver cancer cell metastasis via the toll like receptor 4 (TLR4) signaling pathway^62^. Gut microbiota-stimulated cathepsin K secretion mediates TLR4-dependent M2 macrophage polarization and promotes tumor metastasis in colorectal cancer^63^. These results suggest that the activation of M2 macrophages and related inflammatory immune pathways may worsen AML patient condition and then affect patient prognosis. We also examined the expression levels of the immune checkpoint protein PD-L1 in the two subgroups, and found that the expression level of PD-L1 was increased in cluster1 patients, who showed a decreased OS. This may be because high expression of PD-L1 in AML with increased degree of malignancy promotes the immune escape of leukemia cells and further affects the quality of life in these patients. The abnormal expression of m^6^A-related lncRNAs and the change of immune microenvironment may indicate the condition of AML patients.

The above results indicate that m^6^A-related lncRNAs clustering subgroups were related to the clinicopathologic and biological characteristics of AML. To evaluate the value of these molecules in predicting the prognosis of AML patients, we identified risk signals using LASSO regression analysis, and constructed a risk prediction model based on six lncRNAs to predict OS in AML patients, Lasso regression analysis removes redundant genes, increasing the stability of the model to prevent overfitting. Among the lncRNAs involved in model construction, TRAF3IP2-AS1 is a key regulator of interleukin-17 signaling in autoimmune diseases^64^. Low levels of expression of TRAF3IP2-AS1 promotes the development of non-O-TFE3 translocation renal cell carcinoma^65^. TRAF3IP2-AS1 is a potential biomarker for early diagnosis and prognosis prediction in glioblastoma^66^. In the randomized test set employed in our present study, the OS of patients in the high-risk group was significantly decreased compared with that of patients in the low-risk group. ROC curves, survival curves, and heatmap show that the model possessed good predictive ability. The prediction accuracy of the model was verified using a test set, while consistency of performance was verified in a training set. Univariate and multivariate independent prognostic analyses, and survival analyses, of stratified clinicopathologic factors also show that our model possessed independent predictive ability (independent of age and gender). These results indicate that the risk prediction model showed high reliability, and that the risk score can be used to predict prognosis in AML patients. AML is a hematological tumor, and, thus, lacks a solid tumor-related classification and staging, which are usually used to categorize disease severity and prognosis in patients. Our findings suggest that the risk-scoring model is clinically significant and can be used for prognostic assessment in patients with AML.

Then, we analyzed the characteristics of risk-signaling to show that risk scores were correlated with clinicopathological factors and immune infiltration levels in AML patients, such factors included increased patient age, high immune scores, and high risk scores. The OS of AML patients in cluster1 was relatively short. According to our risk prediction model, the corresponding risk score of cluster1 should have been relatively high. Our results agree with the prediction of the model, thereby indirectly verifying the accuracy of our model in predicting the OS of AML patients. Immune filtration analysis showed that infiltration levels of M2 macrophages, monocytes, and Tregs were positively correlated with the risk score, suggesting that increased numbers of these cells were related to poor prognosis in AML patients. Studies have also shown that interactions between Tregs and M2 macrophages are positively associated with tumor progression^67,68^. As AML progresses, the significant increase in the number of Tregs results in the destruction of immune homeostasis, leading to immune suppression. This is main reason for the immune escape of cancer cells and characteristics of AML progression^69^. Tregs show increased expression of CD14 and CD163, and product C–C motif chemokine ligand 2, thereby promoting the selective transformation of monocytes into M2 macrophages^70,71^. Our correlation analysis of these immune infiltrates yielded results that were consistent with those obtained in previous studies, indicating commonalities in immune-cell infiltration of different tumors. Whether clustering by m^6^A-related lncRNAs or grouping by risk score, we have observed differences in the immune microenvironment of groups with poor prognosis. Based on Toll-like receptors, several TLRs, including TLR 3, have become targets for cancer immunotherapy^72^. The conversion of pro-tumorigenic M2 macrophages to protective M1-phenotype may also be a potential immunotherapy intervention^73^. Reducing the expression of Tregs to relieve its suppression of normal immune cells has also achieved good results^74^. Future studies should utilize the characteristics of relevant infiltrating immune cells to evaluate the prognosis of AML patients, and evaluate these immune cells for targeted therapy in patients with AML.

LncRNAs are involved in regulating the life activities of cells, but most of them need to interact with RNA binding protein (RBP) to play a role^75^. We have observed that TRAF3IP2-AS1 is a regulator of various diseases, and its expression is up-regulated in AML. To study its biological function in AML, we initially explored the RBPs that can be combined with it. Through prediction using an online tool, clinical specimen verification, GEO chip data analysis, and cell experimental research, we found that the RNA-binding protein SRSF10 affects the expression of TRAF3IP2-AS1. AS a member of the SR family of alternative splicing factors, SRSF10 is involved in the post-transcriptional regulation of many genes and the biological processes of various diseases. For example, SRSF10 mediates IL1RAP alternative splicing to regulate the development of cervical cancer^76^, affects BCLAF1 pre-mRNA splicing and regulates the tumorigenic potential of colon cancer cells^77^, and limits the production of HBV RNA^78^. These studies indicate the biological role of SRSF10. Our research found the interaction pair of SRSF10 and TRAF3IP2-AS1. Further regulatory mechanisms and their influence on the development of AML require further experimental research.

In summary, our results indicate that m^6^A-related lncRNAs clustering subgroups are associated with the biological processes of AML and m^6^A-related lncRNAs can be used to predict prognosis in AML patients. Our present research into m^6^A-related lncRNAs represents a novel approach for prediction of AML prognosis. Studying the biological mechanisms of related molecules can also be potentially useful in clinical diagnosis and treatment of AML patients. Our present study shows that m^6^A-related lncRNAs were also associated with the AML tumor immune microenvironment. Immunotherapy has been shown effective in the treatment of refractory AML patients, rendering it a prospective approach in AML therapy. Our study may inspire further development of AML immunotherapy. We also found a correlation pair of SRSF10 and TRAF3IP2-AS1, and further studies may analyze the potential of these two genes in clinical diagnosis and prognostic evaluation. However, the present study also has several limitations. These include its limited sample size and use of samples from only two databases. Future studies should use samples from multiple databases, as well as an increased number of clinical samples, to verify the predictive model established in our present study. Further studies are also needed to fully elucidate the regulatory mechanisms of m^6^A-related lncRNAs in the tumorigenesis and progression of AML at the molecular level.

## Conclusion

In our present study, we show that m^6^A-related lncRNAs were closely related to AML clinical characteristics and tumor immune microenvironment, thereby affecting the tumorigenesis and progression of AML. We also screened for risk-related signaling of m^6^A-related lncRNAs in AML, and constructed and verified a risk prediction model. The results obtained in our present study will aid in the prediction of prognosis and development of immunotherapies in patients with AML.

## Supplementary Information

Supplementary Table 1.
Supplementary Figure 1.

Supplementary Table 1.

Supplementary Table 2.

## Data Availability

All data are available. Please contact us to access if it is needed.
