# Supplementary figures and images for: m6A-related lncRNAs predict prognosis and indicate immune microenvironment in acute myeloid leukemia
Source: Sci Rep. 2022 Feb 2;12:1759. doi: 10.1038/s41598-022-05797-5 (PMC8810799; doi:10.1038/s41598-022-05797-5)

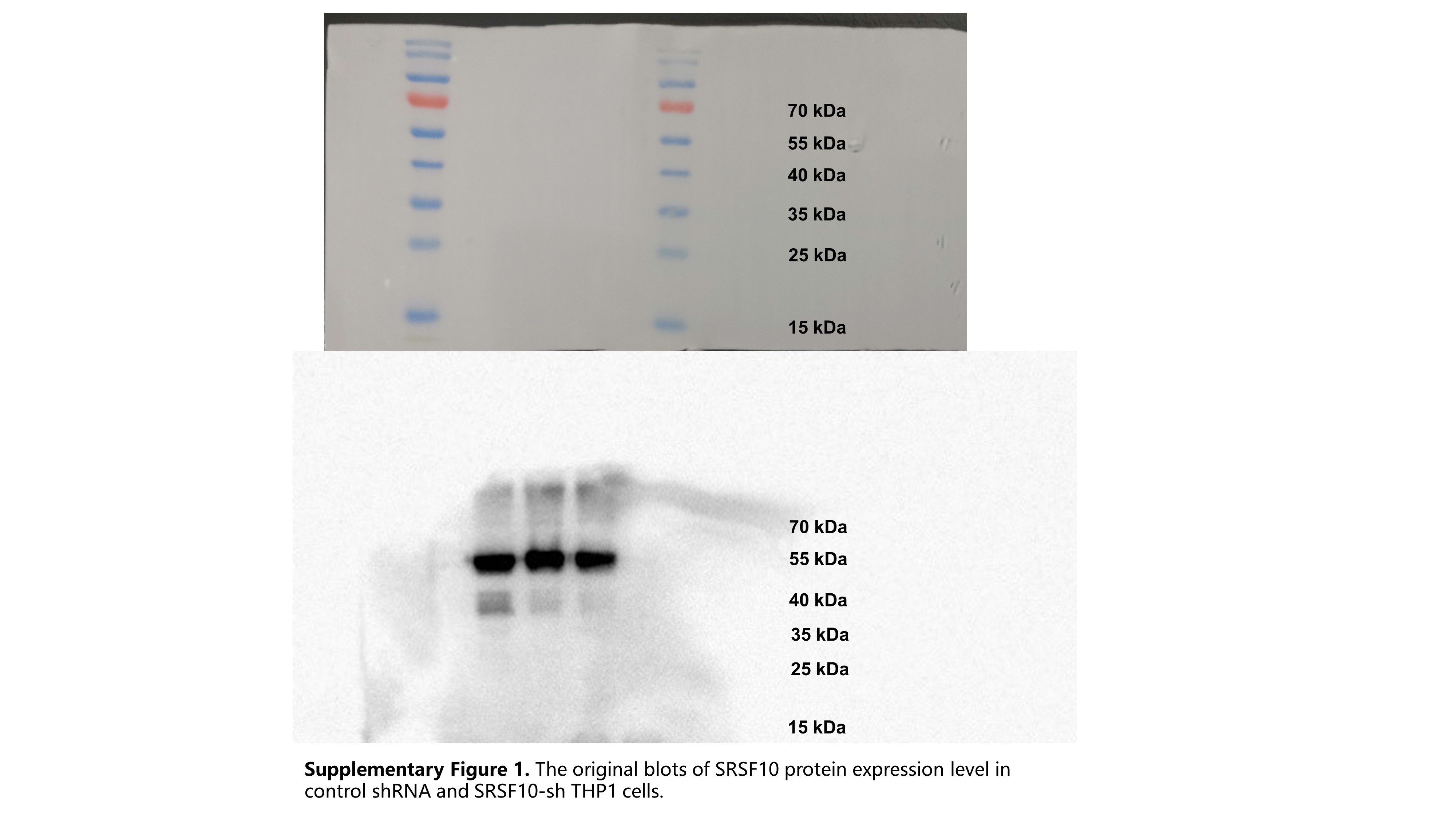

Supplement: Supplementary file 1 — Supplementary Table 1. Supplementary Figure 1. [file 41598_2022_5797_MOESM1_ESM.jpg]
