# Supplementary material for: m6A-related lncRNAs predict prognosis and indicate immune microenvironment in acute myeloid leukemia
Source: Sci Rep. 2022 Feb 2;12:1759. doi: 10.1038/s41598-022-05797-5 (PMC8810799; doi:10.1038/s41598-022-05797-5)
Supplement: Supplementary file 2 — Supplementary Table 1. [file 41598_2022_5797_MOESM2_ESM.docx]

**Supplementary Table 1. Sequences used in this study**

| Primes and siRNA. |  | |  | | Sequences |
| --- | --- | --- | --- | --- | --- |
| GAPDH | | Forward | | 5′-ATGGTGAAGGTCGGTGTGAA-3′ | |
|  |  | Reverse | | 5′-GAGTGGAGTCATACTGGAAC-3′ | |
| SRSF10 | | Forward | | 5′-TGAGGATGTTCGTGATGCTG-3′ | |
|  |  | Reverse | | 5′-CCTTGGCTTTCATCTGATTTGG-3′ | |
| TRAF3IP2-AS1 | | Forward | | 5′-TTTGGCGGCTATGCAGGATT-3′ | |
|  |  | Reverse | | 5′-TGTCCATGTGGTATTGGGCA-3′ | |
| SRSF10-sh1(siRNA) | | Sense | | 5′-GGCGUGAAUUUGGUCGUUATT-3′ | |
|  |  | Antisense | | 5′-UAACGACCAAAUUCACGCGTT -3′ | |
| SRSF10-sh2(siRNA) | | Sense | | 5′-GUGUACAGUUCUUCACGCUTT-3′ | |
|  |  | Antisense | | 5′-AGCGUGAAGAACUGUACACTT-3′ | |
