# Supplementary material for: m6A-related lncRNAs predict prognosis and indicate immune microenvironment in acute myeloid leukemia
Source: Sci Rep. 2022 Feb 2;12:1759. doi: 10.1038/s41598-022-05797-5 (PMC8810799; doi:10.1038/s41598-022-05797-5)
Supplement: Supplementary file 3 — Supplementary Table 2. [file 41598_2022_5797_MOESM3_ESM.docx]

| **Supplementary Table 2. Correlation between m6A regulatory genes and related lncRNAs** | | | | |
| --- | --- | --- | --- | --- |
| **m6A** | **lncRNA** | **cor** | **pvalue** | **Regulation** |
| ALKBH5 | HEIH | 0.527627 | 3.38E-12 | postive |
| METTL3 | CIRBP-AS1 | 0.533292 | 1.79E-12 | postive |
| METTL14 | AL354696.1 | 0.642237 | 6.25E-19 | postive |
| METTL16 | AL354696.1 | 0.510323 | 2.18E-11 | postive |
| ZC3H13 | AL354696.1 | 0.546897 | 3.73E-13 | postive |
| YTHDF1 | AC129492.1 | 0.517772 | 9.89E-12 | postive |
| METTL16 | AL445524.1 | -0.53789 | 1.06E-12 | negative |
| RBMX | AL445524.1 | -0.56713 | 3.15E-14 | negative |
| METTL14 | AC004076.2 | 0.573687 | 1.37E-14 | postive |
| IGFBP2 | MEG8 | 0.501599 | 5.36E-11 | postive |
| METTL3 | AL512343.2 | 0.500969 | 5.71E-11 | postive |
| METTL14 | AP005482.4 | 0.524385 | 4.83E-12 | postive |
| METTL14 | SPAG5-AS1 | 0.576994 | 8.89E-15 | postive |
| IGFBP3 | AC092802.4 | 0.561364 | 6.49E-14 | postive |
| RBMX | LINC00968 | -0.52007 | 7.73E-12 | negative |
| METTL14 | AC020978.3 | 0.546721 | 3.81E-13 | postive |
| RBM15 | AL121832.3 | 0.514524 | 1.40E-11 | postive |
| YTHDC2 | AC012254.3 | 0.606086 | 1.64E-16 | postive |
| METTL3 | AC026362.1 | 0.599715 | 4.07E-16 | postive |
| METTL3 | PAXIP1-AS1 | 0.517414 | 1.03E-11 | postive |
| METTL14 | AC093726.1 | 0.531538 | 2.18E-12 | postive |
| ZC3H13 | AC093726.1 | 0.502126 | 5.08E-11 | postive |
| METTL14 | AC112512.1 | 0.577232 | 8.62E-15 | postive |
| METTL16 | AC112512.1 | 0.51008 | 2.23E-11 | postive |
| RBMX | AL513165.1 | 0.500135 | 6.22E-11 | postive |
| YTHDC1 | AC004492.1 | 0.504376 | 4.03E-11 | postive |
| YTHDC2 | AC004492.1 | 0.589396 | 1.70E-15 | postive |
| FMR1 | AC004492.1 | 0.552889 | 1.83E-13 | postive |
| METTL3 | LINC00653 | 0.562864 | 5.39E-14 | postive |
| METTL14 | AC005332.2 | 0.515101 | 1.32E-11 | postive |
| YTHDC1 | AC005332.2 | 0.520288 | 7.54E-12 | postive |
| METTL3 | TMEM44-AS1 | 0.51923 | 8.45E-12 | postive |
| FTO | AC009005.1 | 0.552016 | 2.03E-13 | postive |
| METTL14 | AC019131.2 | 0.528019 | 3.23E-12 | postive |
| FTO | LINC01107 | 0.878768 | 1.01E-49 | postive |
| FTO | AC004080.2 | 0.554075 | 1.58E-13 | postive |
| METTL3 | AC004908.1 | 0.671837 | 3.64E-21 | postive |
| RBM15 | AC004908.1 | 0.50584 | 3.47E-11 | postive |
| YTHDC2 | AC097634.2 | 0.502339 | 4.97E-11 | postive |
| METTL14 | AC016747.3 | 0.585754 | 2.79E-15 | postive |
| YTHDC1 | AC002553.2 | 0.535171 | 1.45E-12 | postive |
| YTHDF3 | AC002553.2 | 0.50937 | 2.41E-11 | postive |
| METTL3 | LINC00893 | 0.560756 | 7.00E-14 | postive |
| METTL14 | AC005034.6 | 0.562782 | 5.44E-14 | postive |
| LRPPRC | AC005034.6 | 0.559184 | 8.49E-14 | postive |
| RBMX | AC005034.6 | 0.580679 | 5.48E-15 | postive |
| METTL14 | AC023389.2 | 0.599178 | 4.39E-16 | postive |
| ZC3H13 | AC023389.2 | 0.505923 | 3.44E-11 | postive |
| IGFBP3 | LINC00640 | 0.650615 | 1.54E-19 | postive |
| YTHDC2 | AL356805.1 | 0.512267 | 1.78E-11 | postive |
| METTL14 | AL157786.1 | 0.522795 | 5.74E-12 | postive |
| ZC3H13 | AL157786.1 | 0.534253 | 1.61E-12 | postive |
| YTHDC2 | AL157786.1 | 0.539895 | 8.44E-13 | postive |
| FMR1 | AL592424.1 | 0.563728 | 4.84E-14 | postive |
| IGFBP2 | SMC5-AS1 | 0.558918 | 8.77E-14 | postive |
| METTL14 | AC016394.3 | 0.589903 | 1.59E-15 | postive |
| METTL16 | AC016394.3 | 0.530637 | 2.42E-12 | postive |
| YTHDC2 | AC023090.1 | 0.516855 | 1.09E-11 | postive |
| METTL14 | AC004908.3 | 0.511484 | 1.93E-11 | postive |
| RBMX | AC138207.1 | -0.51981 | 7.94E-12 | negative |
| METTL3 | SNHG21 | 0.549806 | 2.64E-13 | postive |
| METTL14 | AL133243.2 | 0.570369 | 2.09E-14 | postive |
| YTHDC1 | AL133243.2 | 0.533297 | 1.79E-12 | postive |
| METTL3 | ZNF436-AS1 | 0.658219 | 4.17E-20 | postive |
| YTHDC1 | AL392172.1 | 0.511727 | 1.88E-11 | postive |
| METTL3 | AC092620.1 | -0.56452 | 4.38E-14 | negative |
| METTL14 | AC124016.3 | 0.571271 | 1.86E-14 | postive |
| FMR1 | FOXN3-AS2 | 0.529819 | 2.65E-12 | postive |
| FTO | MTUS2-AS1 | 0.504335 | 4.05E-11 | postive |
| METTL3 | AL662844.4 | 0.501967 | 5.16E-11 | postive |
| METTL3 | AC004918.5 | 0.521135 | 6.88E-12 | postive |
| METTL14 | CHROMR | 0.533186 | 1.81E-12 | postive |
| METTL3 | AC016394.2 | 0.514079 | 1.47E-11 | postive |
| METTL14 | AC020978.1 | 0.550674 | 2.38E-13 | postive |
| METTL16 | AC020978.1 | 0.505755 | 3.50E-11 | postive |
| RBMX | AC098484.2 | 0.513652 | 1.53E-11 | postive |
| METTL14 | ZNF197-AS1 | 0.559077 | 8.60E-14 | postive |
| METTL16 | AC009120.3 | 0.502637 | 4.82E-11 | postive |
| IGFBP3 | AL136221.1 | 0.512638 | 1.71E-11 | postive |
| METTL14 | AC084824.4 | 0.574028 | 1.31E-14 | postive |
| YTHDC1 | AC084824.4 | 0.541621 | 6.92E-13 | postive |
| METTL3 | AC020907.4 | 0.538231 | 1.02E-12 | postive |
| METTL14 | AC125257.1 | 0.602929 | 2.58E-16 | postive |
| YTHDC1 | AC125257.1 | 0.623028 | 1.32E-17 | postive |
| METTL14 | AC025430.1 | 0.513912 | 1.49E-11 | postive |
| YTHDC2 | AL109923.1 | 0.525685 | 4.19E-12 | postive |
| YTHDC2 | IRF1-AS1 | 0.598645 | 4.73E-16 | postive |
| METTL14 | AC100814.2 | 0.617348 | 3.12E-17 | postive |
| VIRMA | AC100814.2 | 0.563585 | 4.92E-14 | postive |
| ZC3H13 | AC100814.2 | 0.522928 | 5.66E-12 | postive |
| IGFBP2 | AC007383.1 | 0.598406 | 4.89E-16 | postive |
| FTO | AL162274.2 | 0.518318 | 9.33E-12 | postive |
| METTL14 | AC092140.2 | 0.544261 | 5.08E-13 | postive |
| METTL16 | FIRRE | 0.563155 | 5.19E-14 | postive |
| RBMX | FIRRE | 0.538896 | 9.47E-13 | postive |
| METTL14 | AC099811.4 | 0.58442 | 3.33E-15 | postive |
| YTHDC1 | AC099811.4 | 0.627604 | 6.50E-18 | postive |
| FMR1 | AC099811.4 | 0.514834 | 1.35E-11 | postive |
| METTL14 | AC078795.1 | 0.50269 | 4.79E-11 | postive |
| METTL14 | AC021016.2 | -0.53688 | 1.19E-12 | negative |
| FMR1 | AC108058.1 | 0.50959 | 2.35E-11 | postive |
| METTL3 | AC118344.1 | 0.527428 | 3.45E-12 | postive |
| METTL16 | ZNF790-AS1 | 0.50622 | 3.34E-11 | postive |
| IGFBP2 | AC012354.1 | 0.52893 | 2.92E-12 | postive |
| METTL14 | AL596202.1 | 0.501312 | 5.52E-11 | postive |
| YTHDC2 | AL596202.1 | 0.529442 | 2.76E-12 | postive |
| METTL3 | AL391684.1 | 0.52434 | 4.85E-12 | postive |
| YTHDC1 | AL021368.2 | 0.549815 | 2.64E-13 | postive |
| YTHDC2 | AC093423.2 | 0.540495 | 7.88E-13 | postive |
| METTL3 | LINC02021 | 0.502829 | 4.73E-11 | postive |
| RBM15 | RTCA-AS1 | 0.528322 | 3.13E-12 | postive |
| METTL3 | AC004918.1 | 0.532072 | 2.06E-12 | postive |
| METTL14 | AC096586.2 | 0.56498 | 4.14E-14 | postive |
| YTHDC1 | AC096586.2 | 0.546333 | 3.99E-13 | postive |
| YTHDC2 | AC096586.2 | 0.525138 | 4.45E-12 | postive |
| RBMX | FAM157C | -0.50458 | 3.95E-11 | negative |
| VIRMA | AC084024.1 | 0.506032 | 3.40E-11 | postive |
| METTL14 | AC073073.2 | 0.591544 | 1.27E-15 | postive |
| METTL3 | AC005253.1 | 0.519142 | 8.53E-12 | postive |
| METTL14 | OTUD6B-AS1 | 0.558717 | 8.99E-14 | postive |
| VIRMA | OTUD6B-AS1 | 0.714742 | 6.62E-25 | postive |
| ZC3H13 | OTUD6B-AS1 | 0.556641 | 1.16E-13 | postive |
| YTHDF3 | OTUD6B-AS1 | 0.515355 | 1.28E-11 | postive |
| LRPPRC | OTUD6B-AS1 | 0.546227 | 4.04E-13 | postive |
| METTL16 | MIR22HG | -0.50085 | 5.78E-11 | negative |
| IGFBP2 | PCBP3-AS1 | 0.592842 | 1.06E-15 | postive |
| IGFBP3 | AC092802.2 | 0.539643 | 8.69E-13 | postive |
| VIRMA | AC022893.1 | 0.548335 | 3.15E-13 | postive |
| ZC3H13 | AL008729.2 | 0.522508 | 5.93E-12 | postive |
| METTL14 | NNT-AS1 | 0.522781 | 5.75E-12 | postive |
| METTL16 | NNT-AS1 | 0.506189 | 3.35E-11 | postive |
| ZC3H13 | NNT-AS1 | 0.547745 | 3.37E-13 | postive |
| LRPPRC | NNT-AS1 | 0.539635 | 8.70E-13 | postive |
| RBMX | NNT-AS1 | 0.565008 | 4.12E-14 | postive |
| FTO | AC104350.1 | 0.823401 | 1.71E-38 | postive |
| METTL16 | AC107375.1 | 0.530354 | 2.49E-12 | postive |
| YTHDC2 | PAXBP1-AS1 | 0.557673 | 1.02E-13 | postive |
| FMR1 | PAXBP1-AS1 | 0.591849 | 1.22E-15 | postive |
| FTO | AC113349.1 | 0.795353 | 3.28E-34 | postive |
| IGFBP2 | AC009495.3 | 0.703662 | 7.09E-24 | postive |
| METTL3 | AC006435.2 | 0.674144 | 2.37E-21 | postive |
| IGFBP2 | AC007922.2 | 0.526631 | 3.77E-12 | postive |
| METTL14 | AC005288.1 | 0.737747 | 3.30E-27 | postive |
| VIRMA | AC005288.1 | 0.605493 | 1.78E-16 | postive |
| ZC3H13 | AC005288.1 | 0.701285 | 1.16E-23 | postive |
| YTHDC1 | AC005288.1 | 0.555978 | 1.26E-13 | postive |
| METTL14 | AC006480.3 | 0.549776 | 2.65E-13 | postive |
| METTL3 | AL050341.2 | 0.551037 | 2.28E-13 | postive |
| RBMX | EBLN3P | 0.556776 | 1.14E-13 | postive |
| IGFBP2 | AL162231.2 | 0.519599 | 8.12E-12 | postive |
| METTL14 | AC015849.4 | 0.53568 | 1.37E-12 | postive |
| RBMX | AC008753.3 | -0.5127 | 1.70E-11 | negative |
| METTL3 | AL049795.1 | 0.603933 | 2.23E-16 | postive |
| METTL3 | EXTL3-AS1 | 0.504215 | 4.10E-11 | postive |
| METTL14 | AC068620.2 | 0.592325 | 1.14E-15 | postive |
| RBMX | AC068620.2 | 0.502366 | 4.95E-11 | postive |
| YTHDC1 | AL360270.1 | 0.530525 | 2.45E-12 | postive |
| YTHDF1 | AC096577.1 | 0.615789 | 3.94E-17 | postive |
| YTHDC2 | AL390195.3 | 0.522832 | 5.72E-12 | postive |
| IGFBP2 | AFF2-IT1 | 0.554811 | 1.45E-13 | postive |
| METTL3 | SNHG10 | 0.5671 | 3.17E-14 | postive |
| YTHDF1 | MAGI2-AS3 | 0.514588 | 1.39E-11 | postive |
| YTHDC2 | AL118556.2 | 0.523997 | 5.04E-12 | postive |
| FMR1 | AL118556.2 | 0.5005 | 5.99E-11 | postive |
| METTL14 | AC068631.1 | 0.516208 | 1.17E-11 | postive |
| METTL3 | TMCC1-AS1 | 0.507971 | 2.78E-11 | postive |
| LRPPRC | TMCC1-AS1 | 0.509901 | 2.28E-11 | postive |
| METTL14 | MIR302CHG | 0.544582 | 4.90E-13 | postive |
| IGFBP2 | MEG9 | 0.548883 | 2.95E-13 | postive |
| METTL14 | GSTCD-AS1 | 0.54984 | 2.63E-13 | postive |
| FMR1 | ZNF451-AS1 | 0.669153 | 5.94E-21 | postive |
| IGFBP2 | CRNDE | 0.513995 | 1.48E-11 | postive |
| FTO | AP000704.2 | 0.558438 | 9.31E-14 | postive |
| YTHDC1 | ZRANB2-AS1 | 0.507976 | 2.78E-11 | postive |
| LRPPRC | SNHG4 | 0.540464 | 7.91E-13 | postive |
| FTO | AL592430.2 | 0.53998 | 8.36E-13 | postive |
| FMR1 | SOS1-IT1 | 0.534145 | 1.63E-12 | postive |
| METTL3 | LENG8-AS1 | 0.504369 | 4.04E-11 | postive |
| METTL14 | AL731563.4 | 0.536703 | 1.22E-12 | postive |
| ZC3H13 | AL731563.4 | 0.516484 | 1.13E-11 | postive |
| FTO | FOXCUT | 0.69209 | 7.52E-23 | postive |
| METTL3 | LINC00342 | 0.562797 | 5.43E-14 | postive |
| FTO | AL133297.1 | 0.534099 | 1.64E-12 | postive |
| METTL3 | AC232271.1 | 0.606007 | 1.66E-16 | postive |
| LRPPRC | RARA-AS1 | -0.52009 | 7.70E-12 | negative |
| YTHDC1 | LINC02728 | 0.546914 | 3.72E-13 | postive |
| FTO | LINC01619 | 0.564661 | 4.30E-14 | postive |
| METTL3 | AC005670.3 | 0.509466 | 2.38E-11 | postive |
| METTL3 | AL031282.2 | 0.524294 | 4.88E-12 | postive |
| FTO | AL449106.1 | 0.553122 | 1.78E-13 | postive |
| FMR1 | LINC02234 | 0.508374 | 2.67E-11 | postive |
| IGFBP3 | AC002511.1 | 0.535656 | 1.37E-12 | postive |
| YTHDC2 | AC009318.2 | 0.594493 | 8.44E-16 | postive |
| YTHDC1 | AL132989.2 | 0.542534 | 6.22E-13 | postive |
| METTL3 | AC245060.6 | 0.537382 | 1.13E-12 | postive |
| METTL14 | AC012435.3 | 0.51029 | 2.18E-11 | postive |
| METTL16 | AC012435.3 | 0.501304 | 5.52E-11 | postive |
| YTHDC2 | ACAP2-IT1 | 0.562312 | 5.77E-14 | postive |
| YTHDC2 | STARD4-AS1 | 0.511823 | 1.86E-11 | postive |
| METTL14 | AC097376.3 | 0.634524 | 2.18E-18 | postive |
| ZC3H13 | AC097376.3 | 0.514396 | 1.42E-11 | postive |
| METTL14 | AC007406.5 | 0.662793 | 1.86E-20 | postive |
| ZC3H13 | AC007406.5 | 0.603397 | 2.41E-16 | postive |
| FTO | AC027698.1 | 0.860459 | 1.76E-45 | postive |
| METTL14 | OIP5-AS1 | 0.6996 | 1.65E-23 | postive |
| METTL16 | OIP5-AS1 | 0.523937 | 5.07E-12 | postive |
| ZC3H13 | OIP5-AS1 | 0.66042 | 2.83E-20 | postive |
| YTHDC1 | OIP5-AS1 | 0.58184 | 4.70E-15 | postive |
| RBMX | OIP5-AS1 | 0.546457 | 3.93E-13 | postive |
| IGFBP2 | MIR100HG | 0.603252 | 2.46E-16 | postive |
| METTL3 | ZNRD2-AS1 | 0.534507 | 1.56E-12 | postive |
| YTHDC2 | AC027514.2 | 0.54774 | 3.38E-13 | postive |
| METTL14 | LINC00412 | 0.559877 | 7.80E-14 | postive |
| IGFBP2 | AC002368.1 | 0.528639 | 3.02E-12 | postive |
| METTL3 | C1orf220 | 0.541077 | 7.37E-13 | postive |
| IGFBP2 | CACNA1C-IT3 | 0.575694 | 1.05E-14 | postive |
| METTL14 | AC109587.1 | 0.506376 | 3.28E-11 | postive |
| YTHDC2 | AC109587.1 | 0.5279 | 3.28E-12 | postive |
| METTL14 | AC009095.1 | 0.537561 | 1.10E-12 | postive |
| METTL14 | AL132780.2 | 0.523057 | 5.58E-12 | postive |
| FMR1 | LINC00243 | 0.564806 | 4.23E-14 | postive |
| METTL14 | AC110792.3 | 0.638326 | 1.18E-18 | postive |
| YTHDC1 | AC110792.3 | 0.593862 | 9.22E-16 | postive |
| METTL3 | AC068473.5 | 0.536879 | 1.19E-12 | postive |
| METTL3 | MORF4L2-AS1 | 0.634955 | 2.04E-18 | postive |
| METTL14 | AL132780.1 | 0.504393 | 4.03E-11 | postive |
| METTL3 | AC092119.2 | 0.518876 | 8.78E-12 | postive |
| METTL14 | AL356599.1 | 0.541857 | 6.73E-13 | postive |
| IGFBP2 | AL049830.3 | 0.542343 | 6.36E-13 | postive |
| METTL14 | AC011330.2 | 0.598355 | 4.93E-16 | postive |
| YTHDC1 | AC011330.2 | 0.511116 | 2.00E-11 | postive |
| METTL14 | AL590652.1 | 0.526739 | 3.73E-12 | postive |
| YTHDC1 | AL358933.1 | 0.522694 | 5.81E-12 | postive |
| FMR1 | AL133338.1 | 0.523566 | 5.28E-12 | postive |
| METTL3 | AC138956.2 | 0.584773 | 3.18E-15 | postive |
| METTL14 | AC107068.1 | 0.634768 | 2.10E-18 | postive |
| YTHDC1 | AC107068.1 | 0.629011 | 5.22E-18 | postive |
| YTHDC2 | AL357093.2 | 0.514905 | 1.34E-11 | postive |
| METTL14 | AC022726.1 | 0.515492 | 1.26E-11 | postive |
| FMR1 | AP001033.4 | 0.514384 | 1.42E-11 | postive |
| FMR1 | AF127936.1 | 0.500375 | 6.07E-11 | postive |
| METTL14 | AL158825.2 | 0.512124 | 1.80E-11 | postive |
| METTL3 | AL928654.2 | 0.551842 | 2.07E-13 | postive |
| METTL3 | AC009113.1 | 0.532544 | 1.95E-12 | postive |
| METTL3 | AL117336.2 | 0.501763 | 5.27E-11 | postive |
| IGFBP2 | AP003469.2 | 0.512249 | 1.78E-11 | postive |
| METTL14 | AC011477.1 | 0.614482 | 4.79E-17 | postive |
| METTL16 | AC011477.1 | 0.512972 | 1.65E-11 | postive |
| FMR1 | AC242426.2 | 0.714757 | 6.60E-25 | postive |
| IGFBP2 | AP000755.2 | 0.542694 | 6.10E-13 | postive |
| YTHDC2 | AC022893.3 | 0.517234 | 1.05E-11 | postive |
| FMR1 | AC022893.3 | 0.509396 | 2.40E-11 | postive |
| YTHDC1 | AP001486.2 | 0.559916 | 7.76E-14 | postive |
| METTL14 | AC083798.2 | 0.501436 | 5.45E-11 | postive |
| METTL3 | AL158212.3 | 0.561649 | 6.26E-14 | postive |
| METTL14 | AC010300.1 | 0.58259 | 4.25E-15 | postive |
| FTO | AC243571.2 | 0.548257 | 3.18E-13 | postive |
| FMR1 | AC073167.1 | 0.664243 | 1.44E-20 | postive |
| METTL14 | AC012063.1 | 0.559902 | 7.77E-14 | postive |
| RBM15 | LAMTOR5-AS1 | 0.675765 | 1.76E-21 | postive |
| FMR1 | AL034347.1 | 0.511303 | 1.96E-11 | postive |
| METTL14 | AL080317.1 | 0.649242 | 1.95E-19 | postive |
| METTL16 | AL080317.1 | 0.519658 | 8.07E-12 | postive |
| ZC3H13 | AL080317.1 | 0.538179 | 1.03E-12 | postive |
| YTHDC1 | AL080317.1 | 0.527578 | 3.40E-12 | postive |
| METTL14 | ALG13-AS1 | 0.509686 | 2.33E-11 | postive |
| YTHDC1 | ALG13-AS1 | 0.509386 | 2.40E-11 | postive |
| METTL14 | AC099811.5 | 0.512839 | 1.67E-11 | postive |
| YTHDC1 | AC099811.5 | 0.592807 | 1.07E-15 | postive |
| FMR1 | AC099811.5 | 0.503617 | 4.36E-11 | postive |
| YTHDC2 | AC007878.1 | 0.529161 | 2.85E-12 | postive |
| METTL3 | Z97832.2 | 0.500213 | 6.17E-11 | postive |
| METTL14 | AC005261.3 | 0.542173 | 6.49E-13 | postive |
| FTO | AC106738.2 | 0.541022 | 7.41E-13 | postive |
| RBMX | AC092747.4 | 0.534587 | 1.55E-12 | postive |
| ZC3H13 | AP006621.1 | -0.51703 | 1.07E-11 | negative |
| FTO | AC005197.1 | 0.506161 | 3.36E-11 | postive |
| IGFBP2 | AC080037.2 | 0.538843 | 9.53E-13 | postive |
| METTL3 | AC004148.1 | 0.510484 | 2.14E-11 | postive |
| RBMX | AC004148.1 | 0.513409 | 1.57E-11 | postive |
| YTHDC2 | AC006042.3 | 0.506758 | 3.16E-11 | postive |
| FMR1 | AC006042.3 | 0.519274 | 8.41E-12 | postive |
| METTL14 | AC015849.3 | 0.623942 | 1.15E-17 | postive |
| ZC3H13 | AC015849.3 | 0.542989 | 5.90E-13 | postive |
| YTHDC1 | AL606534.1 | 0.512608 | 1.71E-11 | postive |
| YTHDC2 | AL606534.1 | 0.579304 | 6.57E-15 | postive |
| IGFBP2 | LINC02593 | 0.538679 | 9.71E-13 | postive |
| ZC3H13 | AC040169.1 | -0.51142 | 1.94E-11 | negative |
| METTL16 | CKMT2-AS1 | 0.506906 | 3.11E-11 | postive |
| RBMX | CKMT2-AS1 | 0.583434 | 3.80E-15 | postive |
| METTL14 | AC005962.1 | 0.514108 | 1.46E-11 | postive |
| ZC3H13 | AC012467.2 | 0.529102 | 2.87E-12 | postive |
| YTHDC1 | AC084782.3 | 0.501751 | 5.28E-11 | postive |
| METTL3 | MRPL20-AS1 | 0.570242 | 2.13E-14 | postive |
| METTL14 | AL442125.2 | 0.503518 | 4.40E-11 | postive |
| METTL3 | UBA6-AS1 | 0.528092 | 3.21E-12 | postive |
| FTO | AC128707.1 | 0.541747 | 6.81E-13 | postive |
| YTHDC1 | AC131971.1 | 0.52951 | 2.74E-12 | postive |
| FMR1 | AL035409.1 | 0.511855 | 1.85E-11 | postive |
| METTL3 | ZNF32-AS2 | 0.551687 | 2.11E-13 | postive |
| ZC3H13 | AC011477.2 | 0.502269 | 5.00E-11 | postive |
| YTHDC2 | AC107398.2 | 0.514088 | 1.46E-11 | postive |
| METTL14 | AC022558.3 | 0.628561 | 5.60E-18 | postive |
| YTHDC1 | AC022558.3 | 0.52775 | 3.33E-12 | postive |
| IGFBP2 | AP000785.1 | 0.559006 | 8.68E-14 | postive |
| RBM15 | AC253536.3 | 0.529571 | 2.72E-12 | postive |
| METTL3 | AC098484.1 | 0.529863 | 2.63E-12 | postive |
| FTO | AC135178.4 | 0.522829 | 5.72E-12 | postive |
| FMR1 | AC012568.1 | 0.542381 | 6.33E-13 | postive |
| IGFBP2 | LINC01833 | 0.537223 | 1.15E-12 | postive |
| FTO | AC008940.1 | 0.76884 | 9.80E-31 | postive |
| YTHDC2 | AL122035.2 | 0.500203 | 6.17E-11 | postive |
| METTL3 | AL135791.1 | 0.538759 | 9.62E-13 | postive |
| FMR1 | AL359880.1 | 0.51399 | 1.48E-11 | postive |
| METTL14 | AC009961.1 | 0.53842 | 1.00E-12 | postive |
| METTL14 | DNAJC3-DT | 0.591924 | 1.20E-15 | postive |
| ZC3H13 | DNAJC3-DT | 0.530249 | 2.52E-12 | postive |
| METTL14 | AC109361.1 | 0.507525 | 2.91E-11 | postive |
| YTHDC2 | AP002884.1 | 0.527373 | 3.47E-12 | postive |
| METTL16 | AC135178.6 | 0.559929 | 7.75E-14 | postive |
| METTL14 | OCIAD1-AS1 | 0.515416 | 1.27E-11 | postive |
| IGFBP2 | AL161729.1 | 0.503744 | 4.30E-11 | postive |
| METTL3 | AL136368.1 | 0.545188 | 4.56E-13 | postive |
| FTO | AC016642.1 | 0.60248 | 2.75E-16 | postive |
| VIRMA | AC069281.2 | -0.50497 | 3.79E-11 | negative |
| LRPPRC | AC069281.2 | -0.51672 | 1.11E-11 | negative |
| YTHDC1 | AC007014.2 | 0.546434 | 3.94E-13 | postive |
| METTL14 | AC020978.7 | 0.562241 | 5.82E-14 | postive |
| METTL3 | ZNF460-AS1 | 0.510865 | 2.06E-11 | postive |
| YTHDC2 | AC000120.2 | 0.515234 | 1.30E-11 | postive |
| METTL14 | AC245060.5 | 0.578575 | 7.23E-15 | postive |
| ZC3H13 | AC245060.5 | 0.552449 | 1.93E-13 | postive |
| YTHDC1 | AL592148.3 | 0.531899 | 2.10E-12 | postive |
| FMR1 | AL592148.3 | 0.504672 | 3.91E-11 | postive |
| ALKBH5 | AL592148.3 | -0.50574 | 3.51E-11 | negative |
| METTL14 | AC087301.1 | 0.540704 | 7.69E-13 | postive |
| METTL3 | AC011815.1 | 0.522539 | 5.91E-12 | postive |
| FMR1 | AP000866.6 | 0.554527 | 1.50E-13 | postive |
| METTL16 | AC027796.1 | 0.523906 | 5.09E-12 | postive |
| METTL3 | AC174065.1 | 0.528163 | 3.18E-12 | postive |
| YTHDF1 | AL391001.1 | 0.504667 | 3.91E-11 | postive |
| METTL3 | AC048382.2 | 0.504335 | 4.05E-11 | postive |
| YTHDC2 | TMEM161B-AS1 | 0.503986 | 4.20E-11 | postive |
| METTL14 | SEPSECS-AS1 | 0.535358 | 1.42E-12 | postive |
| FMR1 | AL353653.1 | 0.518421 | 9.22E-12 | postive |
| METTL14 | AL354892.3 | 0.550789 | 2.35E-13 | postive |
| METTL14 | AC090425.3 | 0.562157 | 5.88E-14 | postive |
| ZC3H13 | AC090425.3 | 0.516909 | 1.08E-11 | postive |
| METTL14 | AC006213.4 | 0.662823 | 1.85E-20 | postive |
| YTHDC1 | AC006213.4 | 0.557266 | 1.07E-13 | postive |
| FTO | AC009133.3 | 0.567746 | 2.92E-14 | postive |
| YTHDC2 | ITFG1-AS1 | 0.539076 | 9.28E-13 | postive |
| LRPPRC | ZBED3-AS1 | 0.5525 | 1.91E-13 | postive |
| METTL14 | AC004217.1 | 0.517458 | 1.02E-11 | postive |
| VIRMA | AC108449.2 | 0.68218 | 5.22E-22 | postive |
| YTHDC2 | AC108449.2 | 0.515763 | 1.23E-11 | postive |
| FMR1 | AC108449.2 | 0.605267 | 1.84E-16 | postive |
| FMR1 | AL139002.1 | 0.510239 | 2.20E-11 | postive |
| METTL14 | AC034229.4 | 0.501525 | 5.40E-11 | postive |
| IGFBP2 | LINC01513 | 0.580383 | 5.70E-15 | postive |
| RBMX | AC013644.1 | -0.51309 | 1.63E-11 | negative |
| METTL14 | AC007390.1 | 0.603786 | 2.28E-16 | postive |
| YTHDC1 | AC007390.1 | 0.521826 | 6.38E-12 | postive |
| YTHDC2 | AC007390.1 | 0.587785 | 2.12E-15 | postive |
| FMR1 | AC007390.1 | 0.527213 | 3.54E-12 | postive |
| FMR1 | AL078604.2 | 0.528705 | 3.00E-12 | postive |
| METTL14 | AC005225.1 | 0.578155 | 7.64E-15 | postive |
| FTO | AC020916.2 | 0.506738 | 3.16E-11 | postive |
| FMR1 | AP003498.2 | 0.519806 | 7.94E-12 | postive |
| YTHDC1 | AC007431.1 | 0.518202 | 9.44E-12 | postive |
| METTL3 | AC026471.1 | 0.580386 | 5.70E-15 | postive |
| FTO | AC091138.1 | 0.513158 | 1.62E-11 | postive |
| FTO | AC020594.1 | 0.648111 | 2.35E-19 | postive |
| YTHDC1 | AC130650.2 | 0.57906 | 6.78E-15 | postive |
| METTL3 | AC012360.3 | 0.595192 | 7.66E-16 | postive |
| RBMX | AC012360.3 | 0.61699 | 3.29E-17 | postive |
| FTO | AL136018.1 | 0.655975 | 6.16E-20 | postive |
| IGFBP2 | MIR193BHG | 0.682263 | 5.14E-22 | postive |
| METTL3 | AL122010.1 | 0.55931 | 8.36E-14 | postive |
| YTHDC2 | AC073655.2 | 0.504387 | 4.03E-11 | postive |
| METTL14 | AL603839.3 | 0.531424 | 2.21E-12 | postive |
| METTL14 | AC006213.5 | 0.528812 | 2.96E-12 | postive |
| YTHDC1 | AC006213.5 | 0.509117 | 2.47E-11 | postive |
| METTL14 | STX18-AS1 | 0.509801 | 2.30E-11 | postive |
| METTL16 | STX18-AS1 | 0.504424 | 4.01E-11 | postive |
| YTHDC1 | STX18-AS1 | 0.524224 | 4.91E-12 | postive |
| RBMX | STX18-AS1 | 0.50689 | 3.11E-11 | postive |
| YTHDC2 | AC022960.1 | 0.508651 | 2.59E-11 | postive |
| IGFBP2 | AP000755.1 | 0.619839 | 2.14E-17 | postive |
| METTL3 | AC138028.4 | 0.58307 | 3.99E-15 | postive |
| METTL14 | AP000255.1 | 0.529226 | 2.83E-12 | postive |
| METTL14 | AL162734.1 | 0.529679 | 2.69E-12 | postive |
| METTL3 | AC084018.1 | 0.500672 | 5.89E-11 | postive |
| FTO | AC009118.3 | 0.524298 | 4.87E-12 | postive |
| YTHDC2 | AC079921.1 | 0.619389 | 2.29E-17 | postive |
| FMR1 | AC079921.1 | 0.677372 | 1.30E-21 | postive |
| METTL14 | AC134407.1 | 0.544868 | 4.74E-13 | postive |
| METTL14 | GMDS-DT | 0.53966 | 8.68E-13 | postive |
| IGFBP2 | TTLL10-AS1 | 0.583895 | 3.57E-15 | postive |
| METTL3 | AC060780.1 | 0.69507 | 4.14E-23 | postive |
| RBMX | AC060780.1 | 0.506903 | 3.11E-11 | postive |
| IGFBP2 | LINC01645 | 0.710368 | 1.71E-24 | postive |
| METTL14 | AL031716.1 | 0.507991 | 2.78E-11 | postive |
| METTL3 | AL139011.1 | 0.704497 | 5.95E-24 | postive |
| RBM15 | AL139011.1 | 0.550301 | 2.49E-13 | postive |
| IGFBP2 | AC011120.1 | 0.605529 | 1.78E-16 | postive |
| RBM15 | EDRF1-AS1 | 0.548727 | 3.00E-13 | postive |
| YTHDC2 | EDRF1-AS1 | 0.611136 | 7.85E-17 | postive |
| YTHDC2 | FAF1-AS1 | 0.538082 | 1.04E-12 | postive |
| RBMX | AC098869.2 | -0.52174 | 6.44E-12 | negative |
| YTHDC2 | BET1-AS1 | 0.513444 | 1.57E-11 | postive |
| RBM15 | C1RL-AS1 | 0.527175 | 3.55E-12 | postive |
| METTL14 | AL355075.2 | 0.522492 | 5.94E-12 | postive |
| METTL3 | AC004771.2 | 0.555327 | 1.36E-13 | postive |
| METTL14 | AL139353.2 | 0.624932 | 9.84E-18 | postive |
| FMR1 | AL049840.7 | 0.517601 | 1.01E-11 | postive |
| HNRNPC | AC073575.3 | 0.523152 | 5.52E-12 | postive |
| METTL14 | AC025682.2 | 0.613945 | 5.19E-17 | postive |
| METTL14 | AL121655.1 | 0.511028 | 2.02E-11 | postive |
| FMR1 | AC055874.1 | 0.503356 | 4.48E-11 | postive |
| FMR1 | AC010809.3 | 0.539133 | 9.22E-13 | postive |
| METTL14 | AC021851.1 | 0.53177 | 2.13E-12 | postive |
| YTHDC1 | AC021851.1 | 0.50985 | 2.29E-11 | postive |
| METTL14 | AC107027.3 | 0.55206 | 2.02E-13 | postive |
| METTL14 | AL132656.2 | 0.603246 | 2.46E-16 | postive |
| ZC3H13 | AL132656.2 | 0.533944 | 1.67E-12 | postive |
| YTHDC1 | AL132656.2 | 0.547891 | 3.32E-13 | postive |
| FTO | RASSF8-AS1 | 0.561184 | 6.64E-14 | postive |
| METTL3 | AL022341.1 | 0.530688 | 2.40E-12 | postive |
| RBMX | GAS5 | 0.563845 | 4.77E-14 | postive |
| METTL14 | AL158163.1 | 0.503533 | 4.40E-11 | postive |
| YTHDC2 | AL158163.1 | 0.507663 | 2.87E-11 | postive |
| IGFBP2 | AC119403.1 | 0.558967 | 8.72E-14 | postive |
| METTL14 | AL807757.2 | 0.523065 | 5.58E-12 | postive |
| METTL14 | AC025171.3 | 0.544826 | 4.76E-13 | postive |
| YTHDC1 | AC006116.11 | 0.500468 | 6.01E-11 | postive |
| RBMX | LINC01503 | -0.50388 | 4.24E-11 | negative |
| YTHDC2 | AC087854.1 | 0.505391 | 3.63E-11 | postive |
| METTL16 | AC015727.1 | 0.543221 | 5.74E-13 | postive |
| METTL14 | AC104984.6 | 0.52224 | 6.10E-12 | postive |
| METTL16 | AC019069.1 | 0.553951 | 1.61E-13 | postive |
| METTL3 | AL133215.1 | 0.583886 | 3.58E-15 | postive |
| FTO | AC106738.1 | 0.587904 | 2.08E-15 | postive |
| METTL14 | ANKRD10-IT1 | 0.528783 | 2.97E-12 | postive |
| ZC3H13 | ANKRD10-IT1 | 0.544097 | 5.18E-13 | postive |
| YTHDC1 | ANKRD10-IT1 | 0.564041 | 4.65E-14 | postive |
| FTO | DPP10-AS1 | 0.612453 | 6.47E-17 | postive |
| METTL14 | AC009032.1 | 0.537458 | 1.12E-12 | postive |
| YTHDC1 | AC009032.1 | 0.531153 | 2.28E-12 | postive |
| METTL14 | AP000766.1 | 0.624967 | 9.78E-18 | postive |
| YTHDC1 | AP000766.1 | 0.539272 | 9.07E-13 | postive |
| IGFBP2 | LINC01943 | 0.629601 | 4.76E-18 | postive |
| METTL3 | LINC01311 | 0.505522 | 3.59E-11 | postive |
| METTL14 | AC005261.1 | 0.582579 | 4.26E-15 | postive |
| YTHDC1 | AC005261.1 | 0.517564 | 1.01E-11 | postive |
| METTL14 | SP2-AS1 | 0.552912 | 1.82E-13 | postive |
| RBM15 | AL359921.1 | 0.516727 | 1.11E-11 | postive |
| METTL14 | AL354696.2 | 0.608888 | 1.09E-16 | postive |
| ZC3H13 | AL354696.2 | 0.511059 | 2.02E-11 | postive |
| YTHDC1 | AL354696.2 | 0.530699 | 2.40E-12 | postive |
| FMR1 | AC103739.1 | 0.521505 | 6.61E-12 | postive |
| FTO | LINC02385 | 0.680143 | 7.70E-22 | postive |
| IGFBP2 | LINC02675 | 0.714276 | 7.33E-25 | postive |
| METTL3 | AC005306.1 | 0.551791 | 2.08E-13 | postive |
| FTO | AC110285.1 | 0.527779 | 3.32E-12 | postive |
| METTL14 | AC244093.4 | 0.548269 | 3.17E-13 | postive |
| YTHDC1 | AC244093.4 | 0.528965 | 2.91E-12 | postive |
| HNRNPA2B1 | AC244093.4 | 0.521138 | 6.88E-12 | postive |
| METTL14 | ZNF561-AS1 | 0.532026 | 2.07E-12 | postive |
| METTL16 | ZNF561-AS1 | 0.533026 | 1.85E-12 | postive |
| RBMX | ZNF561-AS1 | 0.505562 | 3.57E-11 | postive |
| METTL16 | AC127024.2 | 0.560606 | 7.13E-14 | postive |
| METTL14 | AC127024.4 | 0.623278 | 1.27E-17 | postive |
| METTL16 | AC127024.3 | 0.525157 | 4.44E-12 | postive |
| YTHDC1 | AC015971.1 | 0.555698 | 1.30E-13 | postive |
| FMR1 | AC015971.1 | 0.547514 | 3.47E-13 | postive |
| METTL14 | AC055855.2 | 0.515649 | 1.24E-11 | postive |
| METTL14 | AL158163.2 | 0.581857 | 4.69E-15 | postive |
| YTHDC1 | AL158163.2 | 0.517938 | 9.71E-12 | postive |
| IGFBP2 | SOCAR | 0.64346 | 5.11E-19 | postive |
| METTL3 | TMEM147-AS1 | 0.670043 | 5.05E-21 | postive |
| IGFBP2 | SIX3-AS1 | 0.659136 | 3.55E-20 | postive |
| METTL14 | AC090061.1 | 0.56682 | 3.28E-14 | postive |
| LRPPRC | SNHG16 | 0.518532 | 9.11E-12 | postive |
| RBMX | SNHG16 | 0.583637 | 3.70E-15 | postive |
| FTO | ST8SIA6-AS1 | 0.557729 | 1.02E-13 | postive |
| METTL14 | AP002433.1 | 0.503403 | 4.46E-11 | postive |
| YTHDC2 | AC093157.2 | 0.521327 | 6.74E-12 | postive |
| FTO | AC011444.3 | 0.531012 | 2.32E-12 | postive |
| LRPPRC | AF241728.1 | 0.521182 | 6.85E-12 | postive |
| METTL14 | AL359715.3 | 0.564932 | 4.16E-14 | postive |
| METTL14 | AC011447.3 | 0.52949 | 2.75E-12 | postive |
| METTL3 | AC055855.3 | 0.545387 | 4.46E-13 | postive |
| YTHDC1 | PSMA3-AS1 | 0.578583 | 7.22E-15 | postive |
| YTHDC2 | PSMA3-AS1 | 0.508837 | 2.54E-11 | postive |
| METTL16 | BACE1-AS | 0.507234 | 3.00E-11 | postive |
| METTL3 | TMED2-DT | 0.557031 | 1.11E-13 | postive |
| YTHDC1 | CNNM3-DT | 0.519489 | 8.22E-12 | postive |
| FTO | H1FX-AS1 | 0.537456 | 1.12E-12 | postive |
| METTL14 | ZNF433-AS1 | 0.527601 | 3.39E-12 | postive |
| METTL16 | ZNF433-AS1 | 0.508489 | 2.64E-11 | postive |
| IGFBP2 | AC092159.1 | 0.506241 | 3.33E-11 | postive |
| METTL3 | AC008443.3 | 0.531596 | 2.17E-12 | postive |
| METTL3 | CCDC18-AS1 | 0.547581 | 3.44E-13 | postive |
| METTL14 | AC048382.1 | 0.531665 | 2.15E-12 | postive |
| YTHDC1 | AC048382.1 | 0.561236 | 6.59E-14 | postive |
| VIRMA | AF230666.1 | 0.536028 | 1.32E-12 | postive |
| YTHDC2 | AF230666.1 | 0.588408 | 1.95E-15 | postive |
| YTHDF3 | AF230666.1 | 0.572766 | 1.54E-14 | postive |
| METTL14 | AC008669.1 | 0.51039 | 2.16E-11 | postive |
| VIRMA | AC100821.2 | 0.518591 | 9.06E-12 | postive |
| FTO | RPP38-DT | 0.517143 | 1.06E-11 | postive |
| IGFBP2 | AL591684.2 | 0.7086 | 2.50E-24 | postive |
| YTHDC1 | NARF-IT1 | 0.505398 | 3.63E-11 | postive |
| METTL14 | AC080013.4 | 0.535529 | 1.39E-12 | postive |
| IGFBP2 | AP003469.1 | 0.635205 | 1.96E-18 | postive |
| IGFBP2 | AJ239328.1 | 0.644326 | 4.43E-19 | postive |
| METTL3 | ZNF213-AS1 | 0.617268 | 3.16E-17 | postive |
| YTHDC2 | AC116158.1 | 0.566436 | 3.44E-14 | postive |
| FMR1 | AC116158.1 | 0.573384 | 1.42E-14 | postive |
| METTL14 | AC004158.1 | 0.513932 | 1.49E-11 | postive |
| METTL14 | AC010326.3 | 0.560658 | 7.08E-14 | postive |
| METTL16 | GCC2-AS1 | 0.501285 | 5.53E-11 | postive |
| METTL3 | LINC01089 | 0.518239 | 9.41E-12 | postive |
| RBMX | AC008753.2 | -0.52464 | 4.70E-12 | negative |
| YTHDF3 | AC011978.2 | 0.549579 | 2.71E-13 | postive |
| METTL14 | AC093297.2 | 0.605222 | 1.86E-16 | postive |
| VIRMA | AC093297.2 | 0.500708 | 5.87E-11 | postive |
| ZC3H13 | AC093297.2 | 0.542787 | 6.04E-13 | postive |
| YTHDC1 | N4BP2L2-IT2 | 0.510451 | 2.15E-11 | postive |
| YTHDC2 | N4BP2L2-IT2 | 0.522795 | 5.74E-12 | postive |
| IGFBP2 | CLDN10-AS1 | 0.588865 | 1.83E-15 | postive |
| METTL16 | AC026356.1 | 0.526085 | 4.01E-12 | postive |
| LRPPRC | KTN1-AS1 | 0.516099 | 1.18E-11 | postive |
| METTL3 | MIR4453HG | 0.615183 | 4.31E-17 | postive |
| YTHDC2 | AC084876.1 | 0.501897 | 5.20E-11 | postive |
| FTO | AL357315.1 | 0.667594 | 7.88E-21 | postive |
| METTL14 | AL161756.1 | 0.513035 | 1.64E-11 | postive |
| METTL3 | THUMPD3-AS1 | 0.508017 | 2.77E-11 | postive |
| FTO | PRMT5-AS1 | 0.529989 | 2.60E-12 | postive |
| FMR1 | AP001636.3 | 0.551178 | 2.24E-13 | postive |
| METTL14 | AL021368.1 | 0.506351 | 3.29E-11 | postive |
| METTL16 | AC008280.2 | 0.539141 | 9.21E-13 | postive |
| IGFBP2 | CACNA1C-AS4 | 0.53566 | 1.37E-12 | postive |
| YTHDC2 | SEMA6A-AS1 | 0.571496 | 1.81E-14 | postive |
| IGFBP2 | AC034102.6 | 0.610084 | 9.16E-17 | postive |
| RBMX | AC121338.1 | -0.50456 | 3.96E-11 | negative |
| YTHDC2 | AC116366.2 | 0.527353 | 3.48E-12 | postive |
| IGFBP2 | AL591686.2 | 0.624966 | 9.78E-18 | postive |
| FTO | AP005120.1 | 0.511001 | 2.03E-11 | postive |
| METTL14 | AC005726.4 | 0.635159 | 1.97E-18 | postive |
| METTL3 | AC114730.3 | 0.50003 | 6.28E-11 | postive |
| METTL14 | AC022272.1 | 0.572942 | 1.50E-14 | postive |
| VIRMA | AC022272.1 | 0.516907 | 1.08E-11 | postive |
| METTL14 | HCG18 | 0.510161 | 2.21E-11 | postive |
| METTL16 | HCG18 | 0.518612 | 9.04E-12 | postive |
| IGFBP2 | AJ011931.1 | 0.562547 | 5.60E-14 | postive |
| METTL3 | TTC28-AS1 | 0.583139 | 3.95E-15 | postive |
| METTL14 | AC083806.2 | 0.509547 | 2.36E-11 | postive |
| FTO | AC023301.1 | 0.53189 | 2.10E-12 | postive |
| METTL14 | AC079915.1 | 0.518703 | 8.95E-12 | postive |
| RBMX | AC007728.3 | -0.51269 | 1.70E-11 | negative |
| METTL3 | AC105285.1 | 0.519624 | 8.10E-12 | postive |
| METTL14 | AC004943.3 | 0.505778 | 3.49E-11 | postive |
| RBM15 | AC018653.3 | 0.514222 | 1.44E-11 | postive |
| YTHDC1 | AC025917.1 | 0.554379 | 1.53E-13 | postive |
| YTHDC2 | AC025917.1 | 0.615247 | 4.27E-17 | postive |
| FMR1 | AC025917.1 | 0.608495 | 1.16E-16 | postive |
| IGFBP2 | AL117190.1 | 0.531843 | 2.11E-12 | postive |
| YTHDC2 | AC018752.1 | 0.628416 | 5.73E-18 | postive |
| FMR1 | AC018752.1 | 0.5154 | 1.27E-11 | postive |
| IGFBP2 | AL359091.4 | 0.546853 | 3.75E-13 | postive |
| METTL3 | AC025287.3 | 0.56539 | 3.93E-14 | postive |
| METTL14 | AC105760.1 | 0.512011 | 1.82E-11 | postive |
| FMR1 | LINC02416 | 0.538208 | 1.03E-12 | postive |
| METTL14 | AL391834.1 | 0.501732 | 5.29E-11 | postive |
| METTL3 | AC005104.1 | 0.518493 | 9.15E-12 | postive |
| IGFBP2 | MIR503HG | 0.593248 | 1.00E-15 | postive |
| METTL14 | AC008543.3 | 0.556005 | 1.25E-13 | postive |
| HNRNPC | AP002449.1 | 0.536972 | 1.18E-12 | postive |
| METTL14 | AC006504.7 | 0.520757 | 7.17E-12 | postive |
| METTL16 | AC006504.7 | 0.503623 | 4.36E-11 | postive |
| METTL14 | AC022306.2 | 0.573581 | 1.38E-14 | postive |
| YTHDC1 | AC022306.2 | 0.513335 | 1.59E-11 | postive |
| METTL14 | NORAD | 0.609676 | 9.73E-17 | postive |
| VIRMA | NORAD | 0.536319 | 1.27E-12 | postive |
| YTHDC1 | NORAD | 0.557575 | 1.03E-13 | postive |
| YTHDF3 | NORAD | 0.518844 | 8.81E-12 | postive |
| METTL14 | AC008770.3 | 0.528034 | 3.23E-12 | postive |
| YTHDC1 | AP001350.1 | 0.587302 | 2.26E-15 | postive |
| YTHDF3 | AP001350.1 | 0.510229 | 2.20E-11 | postive |
| METTL3 | RAD51-AS1 | 0.616166 | 3.73E-17 | postive |
| METTL3 | AL162586.1 | 0.535588 | 1.38E-12 | postive |
| METTL14 | LINC00662 | 0.563447 | 5.01E-14 | postive |
| METTL16 | LINC00662 | 0.504426 | 4.01E-11 | postive |
| ZC3H13 | LINC00662 | 0.510848 | 2.06E-11 | postive |
| METTL14 | AC084824.5 | 0.686555 | 2.24E-22 | postive |
| VIRMA | AC084824.5 | 0.501242 | 5.56E-11 | postive |
| ZC3H13 | AC084824.5 | 0.571158 | 1.89E-14 | postive |
| YTHDC1 | AC084824.5 | 0.614121 | 5.05E-17 | postive |
| METTL14 | AC005034.4 | 0.608844 | 1.10E-16 | postive |
| VIRMA | AC005034.4 | 0.537699 | 1.09E-12 | postive |
| ZC3H13 | AC005034.4 | 0.514679 | 1.38E-11 | postive |
| YTHDC1 | AC005034.4 | 0.503329 | 4.49E-11 | postive |
| LRPPRC | AC005034.4 | 0.54881 | 2.97E-13 | postive |
| METTL3 | AC105206.2 | 0.518999 | 8.67E-12 | postive |
| METTL3 | AL139089.1 | 0.501548 | 5.39E-11 | postive |
| METTL14 | AL591767.1 | 0.563323 | 5.09E-14 | postive |
| METTL14 | AC003682.1 | 0.525385 | 4.33E-12 | postive |
| METTL3 | RPARP-AS1 | 0.553175 | 1.76E-13 | postive |
| IGFBP2 | AC005344.1 | 0.567361 | 3.06E-14 | postive |
| METTL14 | AC006141.1 | 0.574662 | 1.20E-14 | postive |
| YTHDC1 | AC006141.1 | 0.535606 | 1.38E-12 | postive |
| METTL3 | AL133367.1 | 0.55322 | 1.75E-13 | postive |
| RBMX | NIFK-AS1 | 0.535999 | 1.32E-12 | postive |
| METTL14 | AC099811.1 | 0.548215 | 3.19E-13 | postive |
| YTHDC1 | AC099811.1 | 0.605903 | 1.68E-16 | postive |
| FMR1 | AC099811.1 | 0.536498 | 1.25E-12 | postive |
| METTL14 | AC004943.2 | 0.532596 | 1.94E-12 | postive |
| METTL14 | AC005332.3 | 0.59068 | 1.43E-15 | postive |
| IGFBP2 | AL731567.1 | 0.573161 | 1.46E-14 | postive |
| IGFBP2 | AC073094.1 | 0.621532 | 1.66E-17 | postive |
| IGFBP2 | MRGPRF-AS1 | 0.698386 | 2.11E-23 | postive |
| YTHDC1 | LINC00624 | 0.53224 | 2.02E-12 | postive |
| YTHDC2 | LINC00624 | 0.500581 | 5.94E-11 | postive |
| YTHDC2 | AL731566.2 | 0.566037 | 3.62E-14 | postive |
| YTHDC2 | AC008906.1 | 0.598076 | 5.12E-16 | postive |
| METTL16 | AC004803.1 | 0.561163 | 6.65E-14 | postive |
| RBMX | AC004803.1 | 0.534606 | 1.55E-12 | postive |
| IGFBP2 | AL133492.1 | 0.518938 | 8.72E-12 | postive |
| METTL3 | AC139256.3 | 0.579105 | 6.74E-15 | postive |
| METTL16 | EIF3J-DT | 0.556351 | 1.20E-13 | postive |
| METTL14 | AC095057.3 | 0.571436 | 1.82E-14 | postive |
| YTHDC1 | AC095057.3 | 0.557273 | 1.07E-13 | postive |
| METTL3 | MYLK-AS1 | 0.560079 | 7.60E-14 | postive |
| IGFBP2 | LINC00997 | 0.532161 | 2.04E-12 | postive |
| IGFBP2 | AC002401.3 | 0.568717 | 2.58E-14 | postive |
| METTL3 | AC026471.4 | 0.602302 | 2.82E-16 | postive |
| METTL14 | AP002812.3 | 0.501977 | 5.16E-11 | postive |
| YTHDC1 | AP002812.3 | 0.501086 | 5.64E-11 | postive |
| FTO | AC012555.2 | 0.538229 | 1.02E-12 | postive |
| METTL14 | AC022558.1 | 0.582388 | 4.37E-15 | postive |
| YTHDC1 | AC022558.1 | 0.506702 | 3.17E-11 | postive |
| METTL14 | DLEU2L | 0.559862 | 7.81E-14 | postive |
| YTHDC1 | DLEU2L | 0.600561 | 3.61E-16 | postive |
| YTHDC2 | FMR1-IT1 | 0.57284 | 1.52E-14 | postive |
| FMR1 | FMR1-IT1 | 0.670729 | 4.46E-21 | postive |
| FTO | LINC02718 | 0.632309 | 3.10E-18 | postive |
| METTL14 | AC130324.1 | 0.607305 | 1.37E-16 | postive |
| METTL16 | AC021422.2 | 0.530772 | 2.38E-12 | postive |
| RBMX | TRAF3IP2-AS1 | 0.519321 | 8.37E-12 | postive |
| METTL14 | AC026356.2 | 0.516698 | 1.11E-11 | postive |
| METTL16 | AC026356.2 | 0.501165 | 5.60E-11 | postive |
| FMR1 | LINC00839 | 0.501687 | 5.31E-11 | postive |
| IGFBP2 | MEG3 | 0.629445 | 4.87E-18 | postive |
| IGFBP2 | AC090515.5 | 0.687299 | 1.94E-22 | postive |
| METTL14 | AC127024.5 | 0.585513 | 2.88E-15 | postive |
| METTL3 | AC245060.2 | 0.576334 | 9.69E-15 | postive |
| RBM15 | AC245060.2 | 0.531733 | 2.14E-12 | postive |
| IGFBP2 | AL136114.1 | 0.620204 | 2.03E-17 | postive |
| METTL14 | AC090739.1 | 0.530991 | 2.32E-12 | postive |
| RBM15 | AC016027.1 | 0.532817 | 1.89E-12 | postive |
| IGFBP2 | LINC00926 | 0.639864 | 9.21E-19 | postive |
| YTHDC2 | LRRC8C-DT | 0.51707 | 1.07E-11 | postive |
| METTL14 | Z83843.1 | 0.527464 | 3.44E-12 | postive |
| YTHDC1 | Z83843.1 | 0.546629 | 3.85E-13 | postive |
| YTHDC2 | Z83843.1 | 0.550287 | 2.49E-13 | postive |
| FMR1 | Z83843.1 | 0.504566 | 3.96E-11 | postive |
| IGFBP2 | AC244502.1 | 0.514782 | 1.36E-11 | postive |
| YTHDC1 | AC073569.1 | 0.502264 | 5.01E-11 | postive |
| FTO | AC022601.1 | 0.562006 | 5.99E-14 | postive |
| METTL14 | AL080317.2 | 0.598874 | 4.58E-16 | postive |
| YTHDC1 | AL080317.2 | 0.538867 | 9.50E-13 | postive |
| METTL14 | AC093227.1 | 0.570433 | 2.07E-14 | postive |
| METTL16 | AC093227.1 | 0.521101 | 6.91E-12 | postive |
| YTHDC2 | AC009318.3 | 0.514831 | 1.35E-11 | postive |
| FTO | AC110285.2 | 0.526685 | 3.75E-12 | postive |
| YTHDC1 | AC092794.1 | 0.620405 | 1.97E-17 | postive |
| RBMX | AC092794.1 | 0.510504 | 2.14E-11 | postive |
| METTL3 | COX10-AS1 | 0.58024 | 5.81E-15 | postive |
